# Orthographic depth and developmental dyslexia: a meta-analytic study

**DOI:** 10.1007/s11881-021-00226-0

**Published:** 2021-05-12

**Authors:** Desiré Carioti, Marta Franca Masia, Simona Travellini, Manuela Berlingeri

**Affiliations:** 1 DISTUM, Department of Humanities, University of Urbino Carlo Bo, Urbino, Italy; 2 Center of Clinical Developmental Neuropsychology, ASUR Marche, Area Vasta 1, Pesaro, Italy; 3 NeuroMi, Milan Center for Neuroscience, Milan, Italy

**Keywords:** Developmental dyslexia, Orthographic depth, Phonological awareness, RAN, Reading skills, Working memory

## Abstract

Cross-cultural studies have suggested that reading deficits in developmental dyslexia (DD) can be moderated by orthographic depth. To further explore this issue and assess the moderating role of orthographic depth in the developmental cognitive trajectories of dyslexic and typical readers, we systematically reviewed 113 studies on DD that were published from 2013 to 2018 and selected 79 in which participants received an official DD diagnosis. Each study was classified according to orthographic depth (deep vs. shallow) and participant age (children vs. adults). We assessed the difference between DD and control groups’ performance in reading tasks and in a wide range of cognitive domains associated with reading (phonological awareness (PA), rapid automatized naming (RAN), short-term working memory (WM), and nonverbal reasoning), including age and orthographies as moderators. We found an age-by-orthography interaction effect in word reading accuracy and a significant effect of age in pseudoword reading accuracy, but we found no effect of age and orthographic depth on the fluency parameters. These results suggest that reading speed is a reliable index for discriminating between DD and control groups across European orthographies from childhood to adulthood. A similar pattern of results emerged for PA, RAN, and short-term/WM. Our findings are discussed in relation to their impact on clinical practice while considering the orthographic depth and developmental level.

**Supplementary Information:**

The online version contains supplementary material available at 10.1007/s11881-021-00226-0.

Developmental dyslexia (DD) “is a specific learning disability that is neurological in origin. It is characterized by difficulties with accurate and/or fluent word recognition and by poor spelling and decoding abilities. These difficulties typically result from a deficit in the phonological component of language that is often unexpected in relation to other cognitive abilities and the provision of effective classroom instruction. Secondary consequences may include problems in reading comprehension and reduced reading experience that can impede the growth of vocabulary and background knowledge.” (International Dyslexia Association, 2002).

Although DD has been extensively studied in the past decades, the debate about its causes has continued because of its multifaceted manifestations (Parrila et al., 2020).

Different theoretical frameworks tried to account for causes of DD by assuming deficit in some domain-general cognitive areas as Phonological Awareness (Bishop & Snowling, 2004; Bradley & Bryant, 1978; Démonet et al., 2004; Gabrieli, 2009; Peterson & Pennington, 2015; Vellutino et al., 2004; Snowling, 1981; Stanovich, 1988; Vellutino, 1979) and Working Memory (see Perceptual Anchoring theory; Banai & Ahissar, 2004, 2010), or by attributing to DD some specific neurobiological dysfunctions in the Magnocellular system (Stein & Walsh, 1997; see Stein, 2019 for a review), or in the cerebellar system (Nicolson et al., 2001; Nicolson & Fawcett, 1990, 2011, 2005; Nicolson et al., 1999). These neurobiological alterations would be at the basis of behavioral deficits observed in DD, like deficits in phonological awareness, visuo-ocular motion, contrast sensitivity (Stein, 2019), and process automation (Nicolson et al., 2001).

In particular, the phonological theory attributes to DD a primary deficit in perceiving and manipulating single linguistic unit sounds (the phonemes) and in accurately relating them to their correspondent graphical representation (the graphemes). Based on empirical studies, this deficit seems to prevail in the spectrum of observed DD behavioral deficits (Ramus et al., 2003; Danelli et al., 2017; Reid et al., 2007), also at a cross-linguistic level (Landerl et al., 2013; Paulesu et al., 2001; Ziegler et al., 2010).

Moreover, according to the double deficit hypothesis (Wolf & Bowers, 1999), phonological awareness would represent just one of the two main cognitive markers of DD. The other one would be the Rapid Automatized Naming (RAN), considered as a phonological task by some authors (Bowey et al., 2005; Clarke et al., 2005; Torgesen et al., 1997), but conceived as independent from phonology by Wolf and Bowers (1999). The idea of co-occurrence of phonological and rapid naming deficit found support in several studies on children and adults (see Parrila et al., 2020 for a review) and in cross-linguistic investigations (Landerl et al., 2013; Ziegler et al., 2010). This is quite surprising if we consider that each language has its proper system of phonological rules. In this perspective, phonological awareness and also RAN (if we focused on the lexical retrieval component) are two linguistic constructs that should be as specific as language and reading. Nevertheless, based on the literature (Landerl et al., 2013; Ziegler et al., 2010; Araújo & Faísca, 2019), they seem to be impaired in DD, although their language-specificity.

In line with the multifaceted spectrum of behavioral deficits that emerged by experimental studies and the wide number of causal theoretical frameworks, Pennington (2006) proposed a multiple cognitive deficit model to describe DD. Some comparative studies (Ramus et al., 2003; Danelli et al., 2017) supported this vision highlighting the number of different cognitive and perceptual deficits associated with dyslexia in different DD patients, and suggesting that they could be moderated by some further variables related to both stages of reading acquisition and language-specific issues.

Currently, DD is widely accepted to manifest differently as a function of age and orthography; a growing body of evidence (Borleffs et al., 2018) has shown that language-specific orthographic codes influence reading difficulties as well as reading acquisition (Seymour, 2005; Seymour et al., 2003; Ziegler & Goswami, 2005; Ziegler et al., 2003).

Nevertheless, to account for cross-linguistic differences was initially hard, as the first age of research on reading in general and on DD, in particular, was characterized by an “Anglo-Saxon bias,” namely, by an extensive volume of evidence from English-speaking countries (Share, 2008; Ziegler et al., 2003). Indeed, among papers on dyslexia between 1990 and 2010, the proportion of those that involve English readers exceeded 50% before 2000, whereas the number of studies in other orthographies increased only in the last 20 years (see Fig. 1). Consequently, most empirical studies and reading models were widely conceived and more suitable for Anglophone participants. Thus, from the late 1990s, the need to revisit all these models in light of orthographic depth became compelling even for neuroimaging issues (Devoto et al., under review; Martin et al., 2016; Martin et al., 2015; Richlan, 2014; Richlan et al., 2011).

**Fig. 1:**
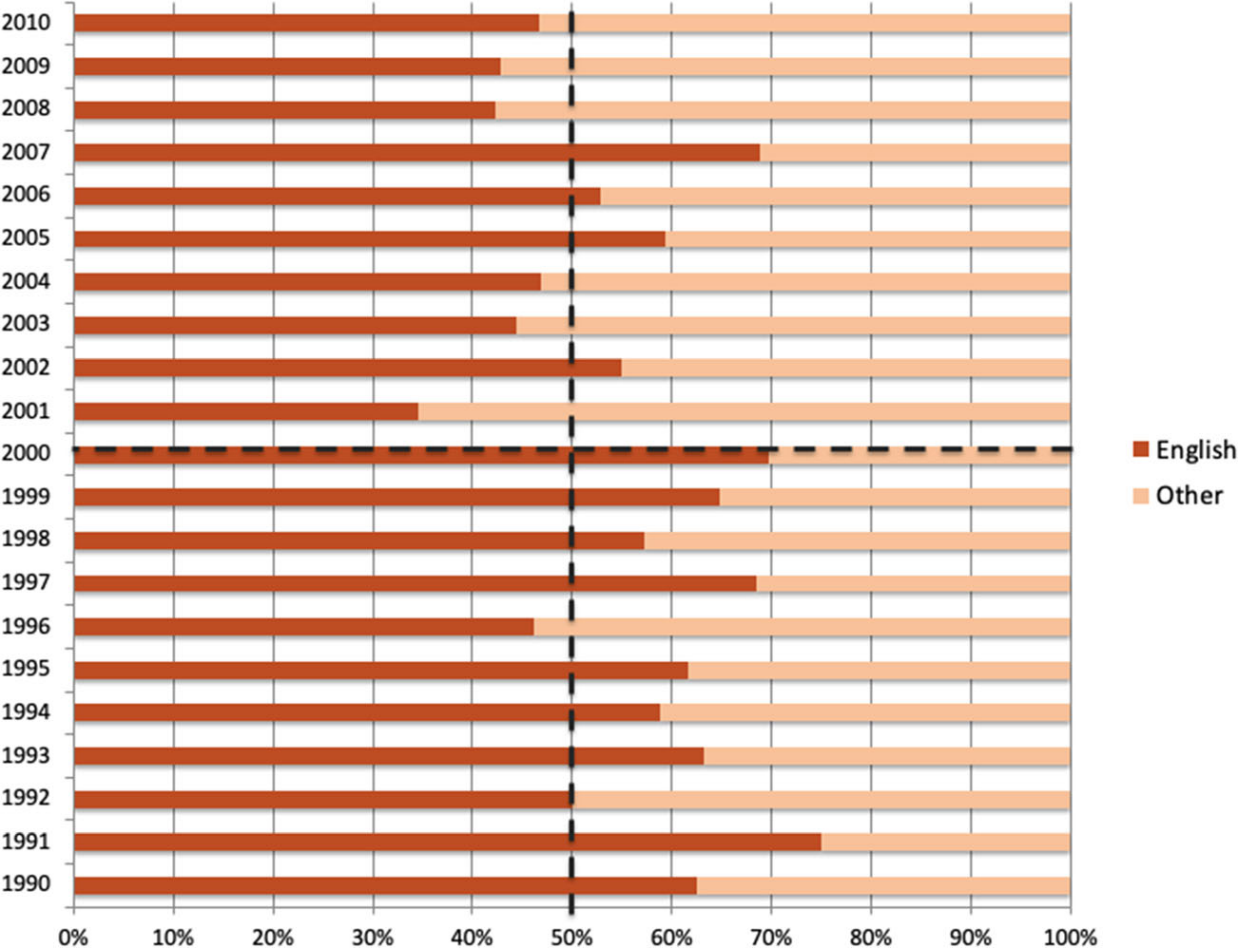
Proportion of English studies published between 1990 and 2010 whose titles included the word “dyslexia.” Before 2000, with only one exception, the percentage of dyslexia studies that include English readers exceeded 50%. Only in the last 20 years, the number of studies published in other languages and orthographies increased, but the percentage of those for English readers always exceeded 30%. This represents the “Anglo-Saxon bias” that characterized the research on the reading process and developmental dyslexia

To investigate the language-specific and universal manifestations of DD, in the last 20 years, some cross-linguistic comparisons have provided a clearer picture of the core cognitive deficits associated with reading disability in childhood and adulthood. Similarly, this meta-analytic study will compare the reading and cognitive skills of non-impaired and dyslexic readers in orthographies characterized by different degrees of consistency. This approach will provide further insights into the nature of reading acquisition differences and the universal character of some common cognitive alterations already indicated as PA and RAN, which are core DD deficits (Georgiou et al., 2008; Landerl et al., 2013; Parrila et al., 2020; Ziegler et al., 2010).

The following section will briefly define (i) developmental trends in DD, (ii) how orthographic consistency influences reading and reading acquisition, and (iii) how orthographic depth shapes the manifestation of DD to introduce the main issues that this meta-analytic study will address.

## Dyslexia in children and adults

As previously highlighted, DD is a developmental disorder that compromises reading efficiency during its acquisition and significantly affects reading proficiency in the life-long period (Scarborough, 1984). The above-mentioned phonological deficit reported by dyslexic children is still found in adults with DD, also when compensation for reading proficiency emerged (Bruck, 1992; Danelli et al., 2017; Elbro et al., 1994; Fostick & Revah, 2018; Olofsson, 2002; Pennington et al., 1990; Ramus et al., 2003; Reid et al., 2007; Scarborough, 1984; Shaywitz et al., 1999; Snowling, 1995; Undheim, 2009).

Indeed, as highlighted by Danelli et al. (2017), magnocellular and motor/cerebellar dysfunctions observed in children are less relevant in adult dyslexic readers (Ramus et al., 2003), while failures in reading fluency and phonological awareness may still be evident (Nergård-Nilssen & Hulme, 2014; see Reis et al., 2020 for a review).

Moreover, according to Eloranta et al. (2019), adults with DD can still exhibit lower proficiency in rapid naming, working memory, and verbal skills. In particular, the rapid naming performance would predict compensational outcomes in adult reading (Eloranta et al., 2019). In a recent meta-analysis by Reis et al. (2020), adult DD readers showed lower performances in several cognitive measures (phonological awareness, working memory, RAN, full IQ, verbal IQ, non-verbal IQ, etc.), although the most severe DD symptoms emerged for reading and writing skills.

These findings dovetail with claims of both phonological theory (Bishop & Snowling, 2004; Bradley & Bryant, 1978; Démonet et al., 2004; Gabrieli, 2009; Peterson & Pennington, 2015; Vellutino et al., 2004; Snowling, 1981; Stanovich, 1988; Vellutino, 1979) and double deficit hypothesis (Wolf & Bowers, 1999), suggesting that phonological awareness and RAN are the more reliable markers of DD, regardless of age, together with some others cognitive aspects as working memory and verbal skills. Reis et al. (2020) showed that orthographic consistency could play a role in moderating DD manifestations in adult readers of deep and shallow orthographies. In line with this, it is even more relevant to consider both developmental and cross-linguistic perspectives when approaching DD.

## Orthographic consistency and reading

Katz and Frost (1992) asserted that a highly stable orthographic system where single-unit sounds and their assembly remain unchanged between words is defined as a “shallow orthography” (see, for example, the Italian word “casa,” where the spelling pattern of the four graphemes corresponds to the pronunciation of the four distinct phonemes [/’kasa/]). By contrast, in some orthographies, certain letters have no phonemic representation (for example, “w” in the English word “whole” [/həʊl/] or “t” in the French word “chat” [/ʃa/]), and some vowels change sounds as a function of the letters they are linked to (for example, the “u” in “ugly” and “huge” in English). Moreover, some decoding rules are generally inconsistent and less stable; these orthographies are referred to as “deep” because the grapheme-to-phoneme correspondence is more “opaque” (Katz & Frost, 1992).

As mentioned above, the different rules that must be implemented across languages and orthographies to obtain a correct and fluent reading process cannot, by definition, allow the application of identical acquisition mechanisms and the manifestation of the same impairment patterns in reading disabilities (Ziegler & Goswami, 2005).

For what concerns accuracy, literacy acquisition studies revealed that readers of shallow orthographies exhibit faster word and nonword reading performance, reaching 80–90% accuracy at the end of the first grade, while readers of deep orthographies suffer from delays in accurate decoding, specifically for nonword reading (Aro & Wimmer, 2003; Frith et al., 1998; Seymour et al., 2003; Seymour, 2005).

According to Seymour et al. (2003), these cross-linguistic differences can be attributed not only to orthographic depth but also to syllabic complexity, a factor that seems to affect in particular fluency and accuracy of nonword reading, that is, the so-called *phonological recoding*. To advance the body of knowledge on language-specific challenges in reading acquisition, Ziegler and Goswami (2005) proposed the *Grain Size Theory*, suggesting that beginner readers encounter three problems: the *availability* of pre-reading phonological and linguistic unit representation, the *consistency* between letters and sounds, and the *granularity* of the orthography.

These works all challenged the validity of the reading models devised for English participants and especially the notion that alphabetic languages maintain a universal switch from a primary alphabetic reading strategy to automatic sight-word reading (Aro, 2004; Ehri, 1987, 1991, 1995, 2005; Frith, 1985).

Children learning to read in shallow orthographies seem mostly dependent on grapheme-to-phoneme conversion to decode words, whereas children learning deep orthographies would benefit more from orthographic whole-word analysis because of the orthographic system’s irregularity (Aro, 2004). Parallel with these predictions, concerning reading fluency, some studies (Ellis & Hooper, 2001; Ellis et al., 2004) reported that younger readers of shallow orthographies have longer latencies when reading longer words while English readers showed no word length effect. Conversely, the latter were more prone to incur substitution errors while readers of shallow orthographies committed nonword reading pronunciation errors more frequently (Ellis et al., 2004). These results suggest that readers of shallow orthographies implement single-phoneme mapping and grapheme-to-phoneme strategies to decode words, while those of deep orthographies depend less on phonological recoding to acquire orthographic representations and lexical storage. This is inconsistent with the idea that the complete acquisition of the alphabetic stage of reading (Ehri & McCormick, 1998; Frith, 1985) is crucial to achieving orthographic analysis; thus, authors such as Castles and Coltheart 2004; Castles et al., 2003) questioned even the idea that phoneme awareness is a cause, rather than a result, of reading acquisition and proficiency in general. Nevertheless, the mutual relation between PA and reading proficiency is widely accepted (Gottardo et al., 2016; Hulme et al., 2005; Perfetti et al., 1987).

## Dyslexia in deep and shallow orthographies: universal and language-specific features

Cross-linguistic studies that explore the language-specific and universal characteristics of DD remain limited; the main challenge to an empirical cross-linguistic comparison of reading is the different methods for evaluating fluency. For example, word and pseudoword reading tasks usually test sight-word reading and phonological recoding, but a different reading fluency measure can be conceptualized via reading assessment in different orthographies; indeed, deep orthographies usually provide an accuracy-based fluency measure that scores participant performance in terms of the number of accurate words read in 45 seconds or 1 minute (e.g., the English Test of Word Reading Efficiency (TOWRE) test; Torgesen et al., 2012). Conversely, shallow orthographies, wherein the ceiling level is more often achieved (Araújo et al., 2015), usually measure fluency using speed (seconds or syllables/seconds) and thus generate one index for accuracy and a separate one for fluency (e.g., the Italian DDE-2 Test; Sartori et al., 2007). Moreover, the overall accuracy level is more often considered by deep orthographies (Share, 2008; Sprenger-Charolles et al., 2011). These methodological differences complicate the direct comparison of performance between, for example, Italian and English readers without adopting ad hoc reading tasks that “parcel out” the specific effects of different procedures and language-specific psycholinguistic variables.

Landerl et al.’s (1997) pioneer study compared reading performance between English and German dyslexic children using 192 words and 192 ad-hoc pseudowords that were similar in spelling and pronunciation and identical in meaning. The authors found that English dyslexics were less accurate and slower in reading pseudowords and were more impaired in reading low-frequency words than their German counterparts. These results indicate a more severe behavioral manifestation of DD in the English context that, as the authors argue, would be “triggered by the key orthographic feature distinguishing German and English orthography, namely the difference in the consistency of grapheme-phoneme relations for vowels” (Landerl et al., 1997, p. 328). Similarly, Ziegler et al. (2003) observed differences between German and English dyslexic readers in overall accuracy but a common impairment in reading speed in all tasks. Notably, both groups reported lower reading fluency levels when compared to age- and level-matched controls but did not perform differently from level-matched controls in terms of accuracy; Ziegler et al. (2003) interpreted this as a delay in accurate reading decoding acquisition. Nevertheless, considering the remarkable difficulty in accurate pseudoword decoding also shown by typical English readers, the authors described this finding as a language-specific feature (Ziegler et al., 2003). This observation is consistent with Paulesu et al.’s (2000) observed behavioral disadvantage for proficient adult readers of deep orthographies and thus seems to represent a language-specific characteristic that remains stable across age. Interestingly, beyond the accuracy results, Ziegler et al. (2003) interpreted the speed-level cross-linguistic deficit as a core DD feature. In particular, DD would be characterized by a “specific nonword reading deficit and a phonological decoding mechanism that operates extremely slowly and serially” (p. 188).

The pattern shown by developmental dyslexic readers in different orthographies was consistent with that reported by typical readers (Landerl et al., 1997; Ziegler et al., 2003). Accordingly, English developmental dyslexic readers reported a lower accuracy than Germans not because of a more severe deficit but because of a generally tougher achievement of reading proficiency in deep orthographies that, in turn, would further exacerbate behavioral symptoms. Within this framework, the first universal behavioral sign of DD would be a reading speed deficit, while, according to the authors, a phonological deficit would represent a cognitive underpinning. Paulesu et al. (2001) reported similar results: despite cross-linguistic differences in reading performance, with Italian readers committing fewer errors for word and nonword reading compared to English and French readers, dyslexic adult readers showed consistent impairment across countries compared to control adult readers. These conclusions are further supported by the meta-analytic results of Reis et al. (2020). Indeed, the most reliable cross-linguistic differences emerged for reading accuracy, in which DD readers of shallow orthographies reported smaller effect sizes when compared to their age-matched controls.

All these findings, when considered with results of behavioral studies on readers of deep (mostly English) orthographies (e.g., Elbro et al., 1994; Griffiths & Frith, 2002; Kemp et al., 2009; Ramus et al., 2003) and more transparent orthographies such as Finnish, Italian, and Spanish (Burani et al., 2006; Laasonen et al., 2012; Leinonen et al., 2001; Suárez-Coalla & Cuetos, 2015), let emerge a clear picture. Dyslexic readers seem to be characterized by a phonological deficit associated with difficulties in implementing an orthographic reading strategy (Ziegler et al., 2008).

Although a reading speed deficit was mainly observed among adult Italian (Burani et al., 2006) and Spanish (Suárez-Coalla & Cuetos, 2015) dyslexic readers, this cannot be considered a language-specific feature of dyslexia because of reading assessment differences and because an accuracy-based reading evaluation is predominant in deep orthographies (Sprenger-Charolles et al., 2011).

Landerl et al.’s (2013) result further supported the hypothesis that dyslexic readers may manifest universal phonological and automation deficits (expressed in terms both of rapid naming and reading fluency; as suggested by Ziegler et al., 2003) since childhood. The authors compared dyslexics and controls from five different orthographies using Seymour et al.’s (2003) five-level classification. They observed that the best predictors of reading deficit were PA and RAN tasks while intelligence quotient (IQ) and short-term/WM played a minor role and that the digit span score predicted the reading process in more inconsistent orthographies (Landerl et al., 2013). Moll et al. (2014) replicated these results on typical readers, finding that PA and RAN were strong predictors of second-graders’ reading skills, with RAN predicting speed and PA accounting for accuracy. These results are also in line with Ziegler et al. (2010), suggesting that PA and RAN are the most reliable reading skill predictors thus far. Once again, this behavioral evidence seems to support the phonological theory (Démonet et al., 2004; Gabrieli, 2009; Peterson & Pennington, 2015; Vellutino et al., 2004; Bishop & Snowling, 2004; Stanovich, 1988; Bradley & Bryant, 1978; Vellutino, 1979; Snowling, 1981) and the double deficit hypothesis (Wolf & Bowers, 1999).

## Research objectives

Several authors (Brislin, 1976; He & Van de Vijver, 2013; Peña, 2007) acknowledged several methodological issues with cross-cultural data collection, most of which were associated with sampling and difficulties in finding adequate cross-linguistic tasks (Landerl et al., 1997; Seymour, 2005). However, the substantial literature on the behavioral and cognitive markers of DD provides a more reliable picture of how orthographic depth affects reading skills development if a meta-analytic approach is employed.

A meta-analytic approach represents a robust method for exploring the universal characteristics of DD by considering the effect of both age and orthographic depth and determining the reliable signatures of DD from findings that are not systematically replicated across studies. This approach enables a more detailed “cognitive characterization” of DD by describing, beyond its universal signs, cognitive characteristics moderated by an interaction between developmental and orthographic factors.

Given this phenomenon’s complexity, we expect different scenarios regarding how DD influences reading accuracy or fluency measures:

- between-groups differences, namely, differences between developmental dyslexic readers and age-matched controls irrespective of age and orthographies, which will help us identify universal cognitive DD markers;
- between-groups differences moderated by age, namely, differences between children and adults with and without DD, which will show variations in the developmental trajectories of reading acquisition across orthographies;
- between-groups differences moderated by orthography, namely, differences between controls and developmental dyslexic readers representing linguistic-specific effects regardless of age;
- between-groups differences moderated by age and orthographic depth, namely, the behavioral differences between developmental dyslexic readers and controls specifically characterizing these groups’ developmental trajectories in a specific linguistic context.

Additionally, we consider other cognitive dimensions, such as RAN, PA, nonverbal reasoning, and short-term/WM, to test whether systematic cognitive differences exist between the two groups and whether they are moderated by our factors of interest.

Finally, this meta-analytic approach will help determine whether the two available reading fluency measures—time-unlimited (TU) and time-limited (TL) tasks—are associated with the same pattern of findings.

## Methods

### Literature search

To compare the reading skills of people with and without DD, from a wide range of European orthographies, we used the US National Library of Medicine’s PubMed online database to search the following keywords: “dyslexia AND test,” “dyslexia AND behavioral/behavioural tasks,” and the more general “dyslexia.”

Due to the high amount of literature about DD, we decided to limit the period meta-analyzed to 5 years. Accordingly, only papers published between 2013 and 2018 were considered. 2013 was considered as a good starting point as it corresponds to the publication year of DSM-5 (American Psychiatric Association, 2013) and, thus, to the updating of diagnostic criteria for learning disorders at an international level, at least for what concerns children.

The first query was run in January 2017; the pool of selected papers was further updated in January 2019.

Initially, we considered 1,994 papers for inclusion, of which 291 were found using “dyslexia AND test,” 59 using “dyslexia AND behavioral/behavioural tasks,” and 1,644 using “dyslexia.” After removing duplicates (*N* = 633), an initial title inspection reduced the selection to 595 studies. Each abstract was reviewed for inclusion criteria and relevance.

### Inclusion and exclusion criteria

We defined inclusion and exclusion criteria based on The Preferred Reporting Items for Systematic Reviews and Meta-Analyses (PRISMA; Moher et al., 2009) and specific requirements imposed by our research questions.

Inclusion criteria limited the selection to studies wherein DD was the main topic. In particular, applied inclusion criteria are reported in follows:

- all participants had official DD diagnoses by a professional neuropsychologist, speech therapist, or other authority in compliance with each country’s provisions;
- an age-matched control group was present;
- the orthographies involved were all European and rated as “deep” or “shallow” according to Seymour et al.’s (2003) classification;
- the results reported clear neuropsychological and cognitive data;
- raw data, with means and standard deviations, were reported or could eventually be calculated based on information about the type of reported scores (T points, C points, standard scores, etc.);
- the type of measure was clearly expressed as, for example, “accuracy,” “proportion of accurate answers,” “total reading time,” or “words read in a minute.”

However, we excluded the following: studies wherein comorbidity with other pathologies was present, even from a neurodevelopmental point of view; studies wherein participants were preschoolers or children with a familial risk of dyslexia; studies conducted in countries where a non-European language is spoken; studies involving poor readers or investigating psychological but not DD-associated cognitive aspects (e.g., quality of life, school satisfaction, and performance anxiety); single-case papers; editorials; reviews and other meta-analyses.

### Literature search results

**Fig. 2:**
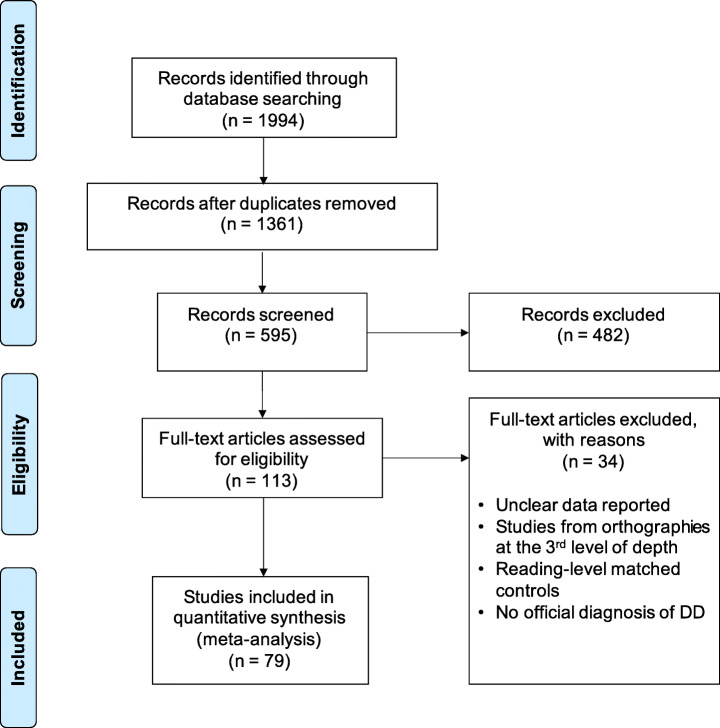
The Preferred Reporting Items for Systematic Reviews and Meta-Analyses (PRISMA) flow diagram (Moher et al., 2009) shows the entire literature recognition and paper selection process. Studies were excluded if they contained incomplete methodological information or met the exclusion criteria

Of the 595 papers selected based on their titles, 113 met the inclusion criteria. Further, we removed 21 studies whose participants read in Portuguese, Dutch, and Swedish: as this study aimed to compare languages with higher transparency and opaqueness, orthographies at the third level of orthographic depth (the medium level per Seymour et al., 2003) were considered confusing and were excluded. From the remaining 92 papers, we extracted behavioral indexes of interest for word reading (accuracy and fluency), non-lexical decoding (accuracy and fluency), RAN, phonologic awareness, short-term/WM, and nonverbal reasoning. Of these, 13 were excluded after a quality check of the methods and reported data, narrowing the sample to 79. Figure 2 shows the selection process.

### Coding procedures

**Fig. 3:**
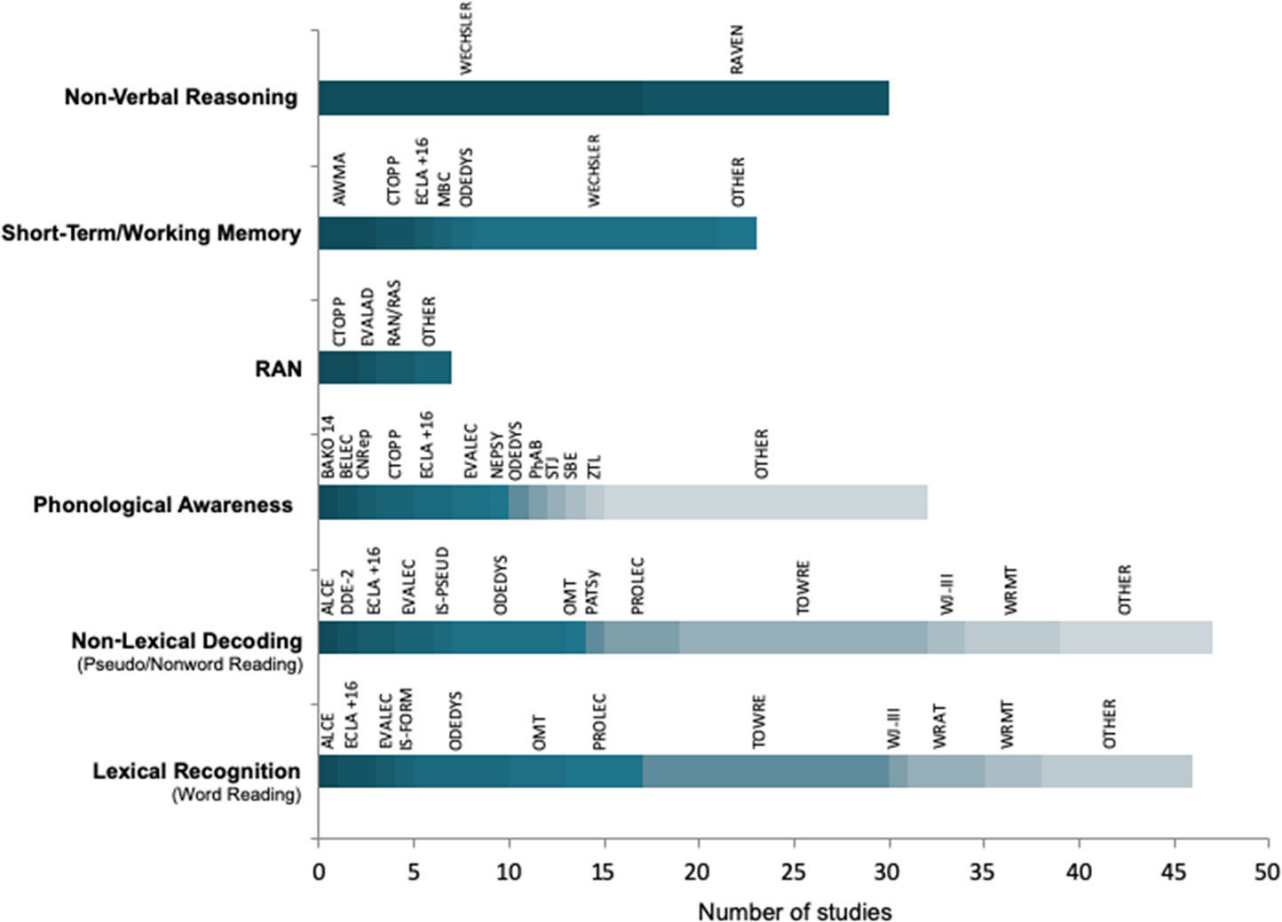
Heterogeneity of tasks and standardized tests available in European countries for each cognitive dimension typically included in the clinical assessment of developmental dyslexia

Different datasets were created for each study dimension based on the information from the papers. The general cognitive dimensions meta-analyzed were word reading, non-lexical decoding, PA, RAN, short-term/WM, and nonverbal reasoning. The first two dimensions helped quantify the reading-associated behavioral deficit in DD across country and age; the last four were used to explore DD-associated cognitive markers and identify universal aspects of this neurodevelopmental deficit. Each dimension was explored by one or more independent meta-analyses depending on the nature of the reported data. For reading measures, for example, we considered two parameters (accuracy and fluency), but we had to address the heterogeneity of adopted measures in each country. In particular, as described in the introduction, reading fluency data are often heterogeneous. Fluency parameters can be measured as total reading time by tests, such as the Spanish PROLEC-R (Cuetos et al., 2007), the Italian DDE-2 battery (Sartori et al., 2007), the French Belec (Mousty & Leybaert, 1999) and ECLA +16 (Gola-Asmussen et al., 2010), or the number of words accurately read in a given time, as in the widely used One Minute Test (Brus & Voeten, 1973), Lecture en Une Minute (Khomsi, 1999), the Dyslexia Screening Test-Junior (Fawcett & Nicolson, 2004), the Ein-Minuten Leseflüssigkeitstest (Willburger & Landerl, 2009), and the TOWRE (Torgesen et al., 2012). The following sections report these two modalities as TU and TL. Figure 3 depicts the heterogeneity of these measures.

Apart from reading fluency, we reported the tests and measures meta-analyzed for each cognitive dimension:

1. *word reading* (including reading high- and low-frequency as well as regular and irregular words, all mainly based on lexical identification), for which three independent meta-analyses for accuracy (total score, percentage, and proportion), TU (sec, msec), and TL (number or percentage of words read) were implemented;
2. *non-lexical decoding* (including nonword and pseudowords reading tests requiring a phonological recoding strategy), for which three independent meta-analyses for accuracy (total score, percentage, and proportion), TU (sec, msec), and TL (number or percentage of words read) were implemented;
3. *phonological awareness*, conceptualized as either *phonological manipulation* (including tasks such as phoneme deletion and elision, segmentation and synthesis, and spoonerisms) or *nonword repetition*, for which all the data in the two meta-analyses concerned accuracy;
4. *RAN*, for which the meta-analysis reported tests on the rapid naming of, mainly, objects, colors, and numbers. Performances were reported in terms of speed (sec, msec);
5. *short-term/WM*, for which the overall meta-analysis primarily reported scores for forward and backward digit span or quotient measures by the WM index of Wechsler’s battery;
6. *nonverbal reasoning*, for which the meta-analysis reported scores from the performance IQ or matrix reasoning subtests of Wechsler’s tests, Raven’s matrices (standard progressive matrices, Raven, 1958; colored progressive matrices, Raven, 2003; Raven, 1956), or other nonverbal reasoning tests as the Cattel Culture Fair Intelligence Test (CCFIT; Cattell, 1940) and the Naglieri Nonverbal Ability Test (Naglieri, 2003).

#### Orthographic depth classification of European orthographies.

Only European orthographies were considered in the present study. In particular, languages and orthographies included were classified as shallow or deep, referring to Seymour’s classification (2003). Here European orthographies were rated at a different depth level based on their grapheme-to-phoneme consistency and rated as “complex” or “simple” based on their syllabic complexity. Seymour’s classification is reported in Table 1. As widely supported in literature (see Schmalz et al., 2015 for a review) and according to Seymour’s classification, English represents the deeper and more complex level.

**Table 1:** Seymour’s classification of European Orthographies (Seymour et al., 2003, p. 146)

		*Orthographic Depth*				
		Shallow				Deep
		1st level	2nd level	3rd level	4th level	5th level
*Syllabic Complexity*	*Simple*	Finnish	Greek Italian Spanish	Portuguese	French	English
	*Complex*		German Norwegian Icelandic	Dutch Swedish	Danish	

#### Factor and variable coding.

**Fig. 4:**
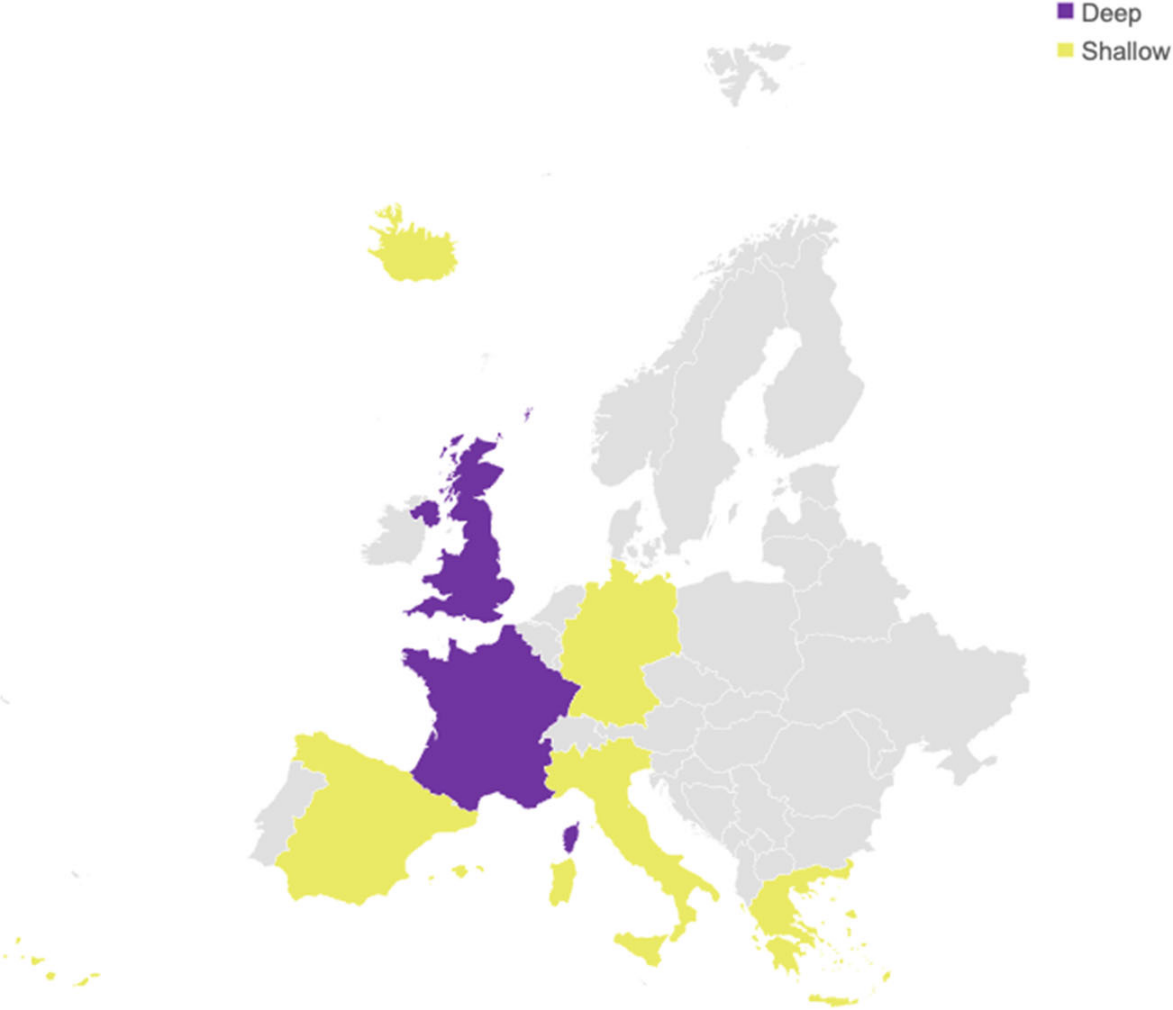
Representation of European countries involved in the meta-analysis. Countries in purple adopt a deep orthography; those in yellow represent countries that implement a shallow orthography. Sixteen further studies included were conducted in the US, and one additional research was done in Australia; all were categorized as “deep orthography”

From each selected paper, we extracted cognitive dimensions, specific tasks within each dimension, and the type of raw measure associated with each task. For example, from Cantiani et al. (2015), we extracted the “phonological awareness” dimension, the “phonological manipulation” task, and the corresponding “accuracy” measure. Each task’s mean raw score and standard deviation were reported for both the dyslexia group (DG) and the control group (CG), with each group’s mean age (expressed in years). Subsequently, we re-coded age into a dichotomous variable: children (8–13 years) and adults (19–32 years). Moreover, for each measure, we also included whether the participants’ countries adopted a shallow or deep orthography according to Seymour et al.’s (2003) classification of European orthographies (see Table 1). Specifically, the shallow level included only measures extracted from studies in which the orthography was at level 1 or 2; the deep level corresponded to levels 4 and 5. See Fig. 4 and Table S1 for more details about orthographies of papers included.

### Statistical analysis

#### Effect size calculation

The analyses were conducted in R (R Core Team, 2015) with the “metafor” package (Viechtbauer, 2010). The effect size of each measure, namely, standardized differences between DG and CG, was calculated using the *escalc* function. If multiple measures using the same cognitive dimension were reported within a single paper (e.g., high- and low-frequency word reading accuracy in the same participant samples), then a single effect size was computed using the *agg* function for the aggregation of dependent effect sizes in the R package “MAd” (Del Re & Hoyt, 2014).

#### Data analysis

First, we identified outliers by exploring the boxplots and calculating the *I*^2^ index (Higgins et al., 2003) using the *rma* function and computing a random effect model (REML). Second, we ran a second REML to check whether between-studies heterogeneity was reduced and decide whether to conduct a further REML including the categorical variables “age” and “orthography” as moderators or to run a fixed effect (FE) model without any further moderator (only when the *tau* value is 0). Finally, we selected the best-fit model based on the Aikake information criterion, a widely used model selection standard (Arnold, 2010).

Moreover, to check whether effect sizes of nonverbal reasoning would predict variations in effect sizes of word reading (accuracy, time unlimited, and limited), we run three further meta-regressions. Only studies that provide data for both nonverbal reasoning and word reading accuracy and fluency (TU and TL) were included in the meta-regressions.

## Results

This meta-analytic study included 79 articles including 450 ESs comparing dyslexic with age-matched typical readers. The total sample size was 14,947 participants, 49% (mean sample size = 659.4; SD = 209.4) for DG and 51% (mean sample size = 682.6; SD = 206.12) for CG. In all meta-analyses the DG (mean age_(adults)_= 23.54, SD = 2.88; mean age_(children)_ = 10.22, SD = 0.77) and CG (mean age_(adults)_ = 23.69, SD = 3.76; mean age_(children)_ = 9.91, SD = 0.72) groups were equivalent in age (as reported in Table S3 of supplementary materials).

Overall, for what concerns the orthographic depth, 67.4% of studies included were rated as “deep” and 32.5% as “shallow,” providing further evidence in favor of the Anglo-Saxon bias.

In what follows, results of models run on each reading and cognitive dimension are reported. We reported only the best-fit model results for each dimension together with the ensuing heterogeneity level, the main effect, or the interaction effect (of moderators, in the case of REML) when significant. Moreover, tables report the number of studies for each moderator and the average effect sizes in each model.

### Word reading

#### Accuracy

This study initially reported 59 observations for both DG and CG in the word reading accuracy dataset. Of these, 36 were dependent and were thus aggregated through the *agg* procedure, resulting in 41 total observations extracted from 38 studies (Casini et al. (2018) reported results from two experiments, Nittrouer and Lowenstein (2013) reported results from two different samples for both DG and CG, and Ruffino et al. (2014) reported data of two different samples of DD readers with and without phonological deficit ).

**Fig. 5:**
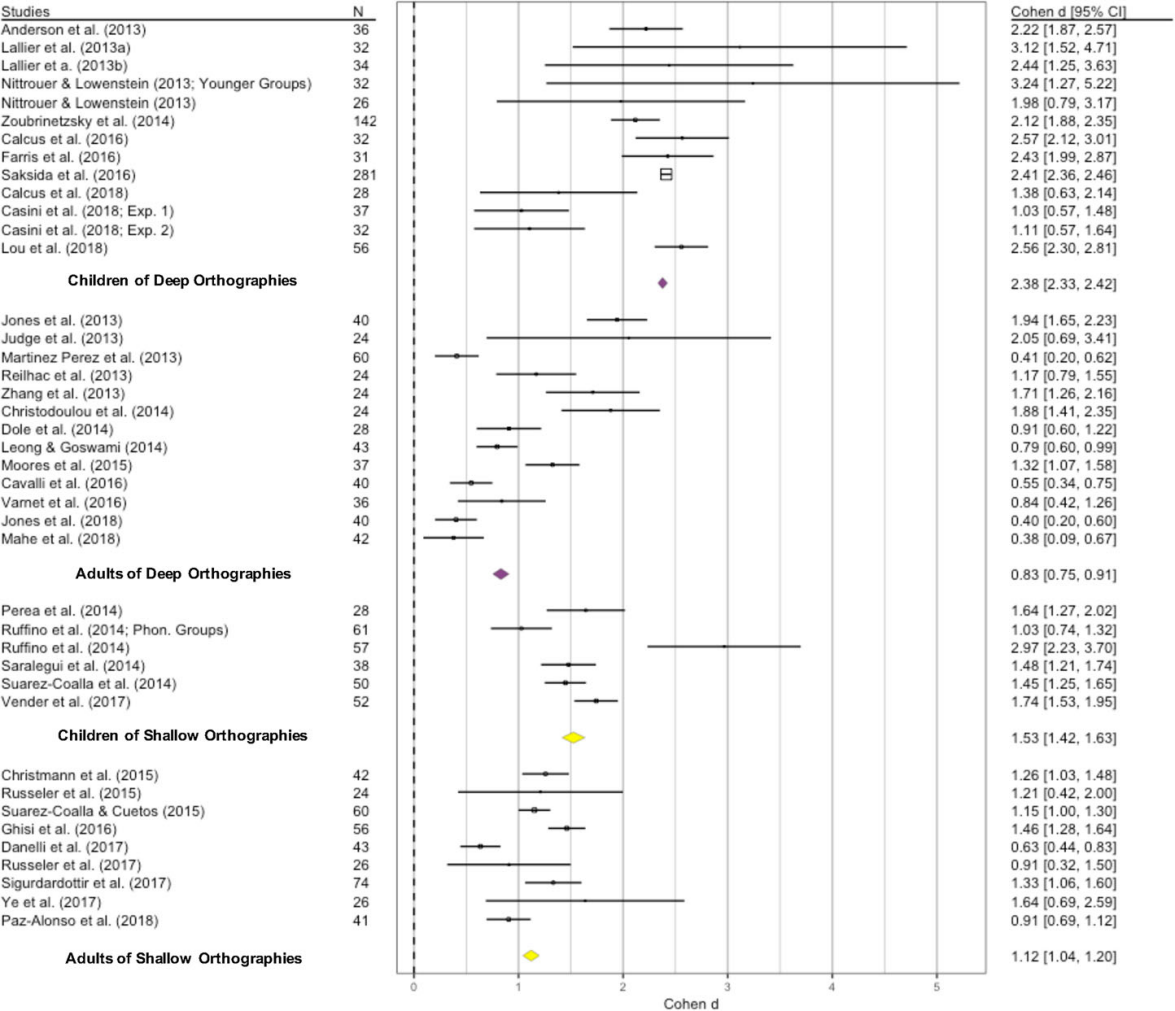
Forest plot for the meta-analysis of the 41 word reading accuracy observations. The average effect size associated with each group (adults and children under deep and shallow orthographies) is represented by diamonds. The yellow diamonds indicate the average effect size for shallow orthographies; the purple ones refer to deep orthographies

The best-fit model for the data was the REML with “orthography” and “age” as moderators (see supplementary materials for model selection details). The residual heterogeneity test was significant (QE_(37)_ = 56.5, *p* = 0.02; *I*^2^ = 32.63%, tau^2^ = 0.07, SE = 0.05), and the same can be said for the moderators’ test (QM_(3)_ = 39.3, *p* < 0.001). The model, which stratified the studies based on the two moderators (see Table 2), revealed a significant difference between DG and CG (*Z* = 14.34, SE = 0.15, *p* < 0.001), and an age-by-orthography interaction (*Z* = 2.24, SE = 0.32, *p* = 0.02). In particular, as summarized in Table 3 and Fig. 5, children of shallow orthographies reported smaller effect size (1.53, 1.42–1.63) than those of deep (2.38, 2.33–2.42) and, accordingly, the increasing trend between children and adult was broader in deep (from 2.83 to 0.83) than in shallow orthographies (from 1.53 to 1.12).

**Table 2:** Distribution of the observations in each reading and cognitive dimension considered, type of model run, and average Effect Sizes (ESs) extracted

	***Word Reading***							
	**Orthographic Depth**	**Age**	**Records (k)**	**Participants (N)**	**Model**	**Average ES**	**min.**	**max.**
*Accuracy*	Shallow	Children	6	286	REML with moderators	1.53	1.42	1.63
		Adults	9	392		1.12	1.04	1.20
	Deep	Children	13	799		2.38	2.33	2.42
		Adults	13	461		0.83	0.75	0.91
		Tot.	41	1938				
*Fluency (TU)*	Shallow	Children	3	116	REML			
		Adults	4	186				
	Deep	Children	4	488				
		Adults	8	296				
		Tot.	19	1086		-1.47	-1.50	-1.44
*Fluency (TL)*	Shallow	Children	3	180	REML			
		Adults	2	130				
	Deep	Children	11	362				
		Adults	8	279				
		Tot.	24	951		1.82	1.76	1.88
	***Non-Lexical Decoding***							
	**Orthographic Depth**	**Age**	**Records (k)**	**Participants (N)**	**Model**	**Average ES**	**min.**	**max.**
*Accuracy*	Shallow	Children	6	289	REML with moderators			
		Adults	9	392				
	Deep	Children	7	581				
		Adults	16	552				
	Regardless of Orthographic Depth	Children	13	870		1.97	1.94	2.01
		Adults	25	944		1.23	1.18	1.28
		Tot.	38	1814				
*Fluency (TU)*	Shallow	Children	5	228	REML			
		Adults	3	143				
	Deep	Children	4	485				
		Adults	8	306				
		Tot.	20	1202		-1.40	-1.43	-1.37
*Fluency (TL)*	Shallow	Children	4	204	REML with moderators			
		Adults	2	132				
	Deep	Children	8	263				
		Adults	7	245				
		Tot.	21	844		2.01	1.95	2.08
	***Phonological Awareness***							
	**Orthographic Depth**	**Age**	**Records (k)**	**Participants (N)**	**Model**	**Average ES**	**min.**	**max.**
*Phonological Manipulation*	Shallow	Children	4	195	REML			
		Adults	3	144				
	Deep	Children	13	836				
		Adults	17	556				
		Tot.	37	1731		1.19	1.16	1.21
*Nonword repetition*	Shallow	Children	6	372	REML			
		Adults	3	125				
	Deep	Children	8	292				
		Adults	5	163				
		Tot.	22	952		1.12	1.07	1.17
	***Other Cognitive Skills***							
	**Orthographic Depth**	**Age**	**Records (k)**	**Participants (N)**	**Model**	**Average ES**	**min.**	**max.**
*RAN*	Shallow	Children	2	99	REML			
		Adults	2	85				
	Deep	Children	8	570				
		Adults	15	490				
		Tot.	27	1244		-1.05	-1.11	-0.99
*Short-Term/WM*	Shallow	Children	5	222	REML			
		Adults	3	140				
	Deep	Children	8	503				
		Adults	14	431				
		Tot.	30	1296		0.84	0.79	0.88
*Non-Verbal Reasoning*	Shallow	Children	8	384	FE			
		Adults	9	380				
	Deep	Children	12	442				
		Adults	20	683				
		Tot.	49	1889		0.19	0.16	0.22

**Table 3:** Results of the REML with moderators run on word reading accuracy, non-lexical decoding accuracy and fluency (TL)

Word Reading							
Accuracy		estimate	se	z	p	CI. lb	CI. ub
	*Intercept*	2.17	0.15	14.34	**< .001**	1.87	2.46
	*Age*	-1.19	0.20	-5.87	**< .001**	-1.58	-0.79
	*Orthography*	-0.57	0.24	-2.31	**0.02**	-1.05	-0.08
	Orthography* Age	0.72	0.32	2.24	**0.02**	0.09	1.35
Non-Lexical Decoding							
Accuracy		estimate	se	z	p	CI. lb	CI. ub
	*Intercept*	2.05	0.16	12.39	**< .001**	1.73	2.38
	*Age*	-0.77	0.20	-3.8	**< .001**	-1.18	-0.37
	*Orthography*	-0.03	0.25	-0.13	0.89	-0.52	0.45
	Orthography* Age	0.17	0.31	0.53	0.58	-0.44	0.79
Fluency (TL)	*Intercept*	2.69	0.25	10.61	**< .001**	2.19	3.18
	*Age*	-0.38	0.36	-1.06	0.28	-1.08	0.32
	*Orthography*	-0.66	0.41	-1.62	0.1	-1.47	013
	Orthography* Age	0.31	0.64	0.48	0.62	-0.94	1.56

Significant effects are highlighted in bold

#### Fluency—time unlimited (TU)

We obtained 19 observations from 19 studies for word reading TU by aggregating six dependent measures from the 25 initial observations in the dataset.

**Fig. 6:**
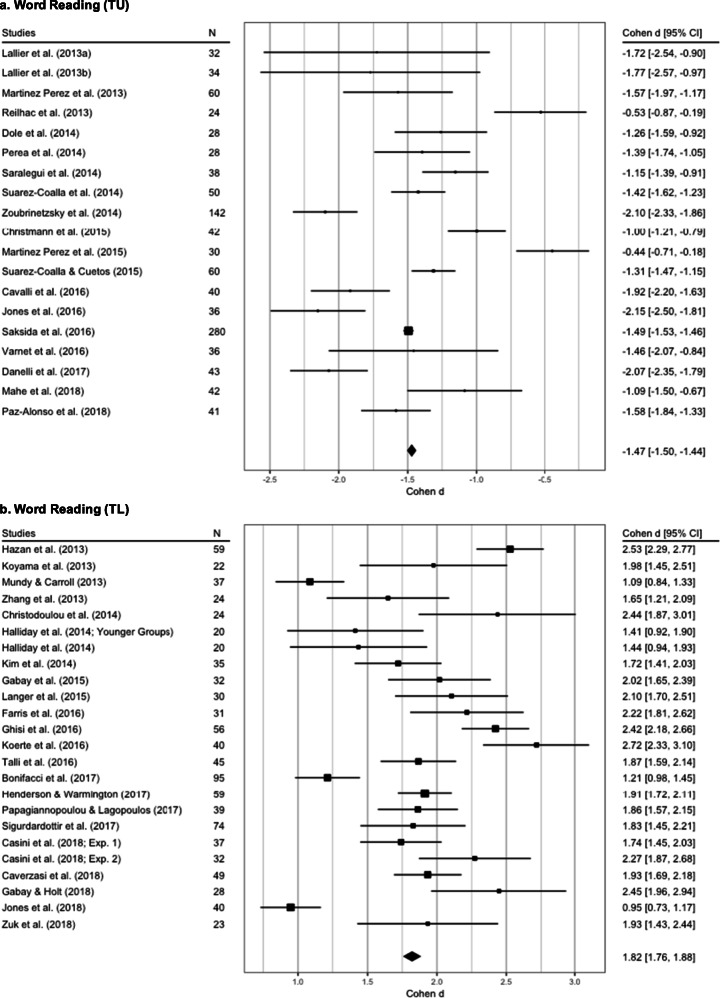
Forest plots of the word reading fluency meta-analyses. The average effect size for each fluency variable (either time limited (TL) or time unlimited (TU)) is represented by the black diamond. Panel **a**: effect size of studies wherein the recorded measure of fluency was TU. Panel **b**: effect size of studies where the recorded measure of fluency was TL, i.e., the number of correct words read in a limited time

The REML without moderators was the best-fit model (see supplementary materials for more details) and revealed a significant between-group difference (*Z*=−13.43, SE =0.10, *p* < 0.001; see Fig. 6, panel a, and Table 2 for more details) with a nonsignificant level of heterogeneity (*Q*_(18)_= 27.26, *p* = 0.07, *I*^2^ = 37.44%, tau^2^ = 0.07, SE = 0.06).

#### Fluency—time limited (TL)

First, we aggregated the dependent measures from Sigurdardottir et al. (2017) and Bonifacci et al. (2017) in single effect size, with the final dataset including 25 studies with 26 observations (Casini et al. (2018) and Halliday et al., (2014)  reported measures from two different dyslexic and control samples). After exploring the boxplots, we rated Lallier et al. (2013c) and Bogon et al. (2014) as outliers and removed them. Thus, the first REML was implemented on 24 observations extracted from 23 papers.

The best-fit model for the data was the REML without moderators (see supplementary materials for details about model selection). The model revealed a nonsignificant level of heterogeneity (*Q*_(23)_= 32.11, *p* = 0.09, *I*^2^ = 32.23%, tau^2^ = 0.07, SE = 0.07) and a significant between-group difference (*Z*=18.32, SE =0.1, *p* < 0.001; see Fig. 6, panel b, and Table 2 for more details).

### Non-lexical decoding

#### Accuracy

The dataset initially included 40 observations, but after exploration of the boxplot, we identified 2 outliers (Asbjørnsen et al. (2014) and the group without a phonological deficit in Ruffino et al. (2014)) and removed them from the dataset. The final dataset included, thus, 38 observations.

The best-fit model included the moderators “age” and “orthography” (see the supplementary materials for more details on model selection).

**Fig. 7:**
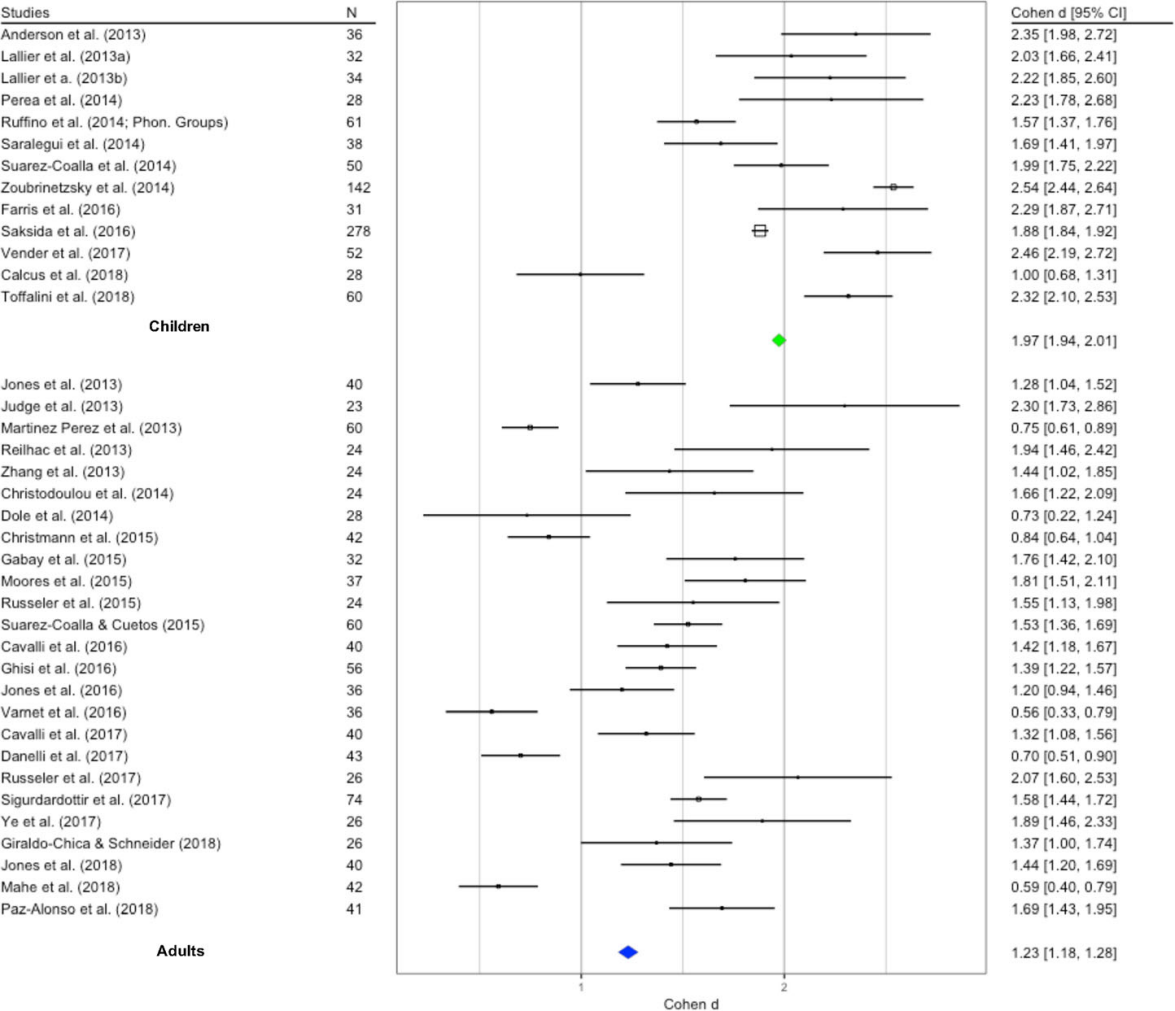
Forest plot of the random effect model used on non-lexical decoding accuracy. The average effect size associated with each participant group (adults and children under deep and shallow orthographies) is represented by diamonds. The green diamond shows the average effect size for children; the blue one indicates adult readers

The residual heterogeneity test was significant (QE_(34)_ = 55.21, *p* = .01; *I*^2^ = 38.9%, tau^2^ = 0.07, SE = 0.05); an overall significant effect of the moderators was observed (QM_(3)_ = 21.36, *p* < 0.001). Table 2 shows the stratification of the studies. In particular, we found a significant between-groups difference (*Z* = 12.39, SE = 0.16, *p* < 0.001), a main effect of age (*Z* = −3.8, SE = 0.20, *p* < 0.001), but no effects of orthography (*Z* = −0.13, SE = 0.25, *p* = 0.89) nor of interaction (*Z* = 0.53, SE = 0.31, *p* = 0.58) emerged (see Table 3 and Fig. 7). Accordingly, we observed an effect size of 1.97 (1.94–2.91) for children and 1.23 (1.18–1.28) for adults, regardless of orthography.

#### Fluency—time unlimited (TU)

**Fig. 8:**
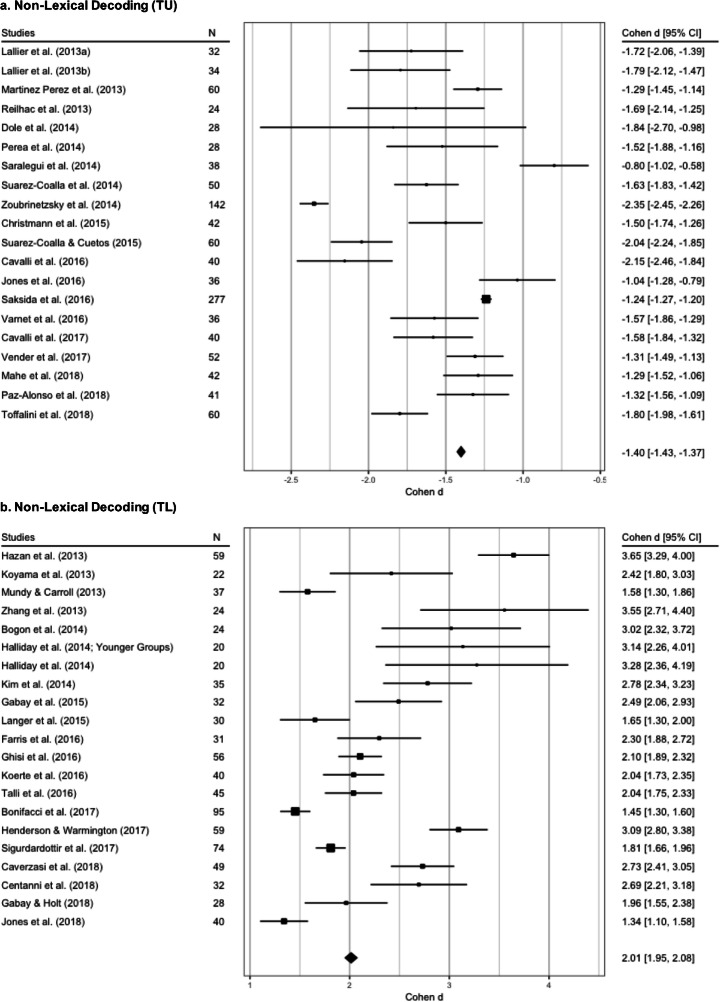
Forest plots of meta-analyses performed on non-lexical decoding fluency. The average effect size for each fluency variable (either time limited (TL) or time unlimited (TU)) is represented by the black diamond. Panel **a**: effect size of studies wherein the fluency measure was recorded as TU. Panel **b**: effect size of studies where the recorded measure of fluency was TL, i.e., the number of correct pseudo/nonwords read in a limited time. Notably, Halliday et al. (2014) reported results from different groups of dyslexics and controls

We rated one study (Danelli et al., 2017) as an outlier through the boxplot exploration and removed it from the dataset. Moreover, 2 observations from the same study (Dole et al., 2014) were aggregated through the *agg* procedure, obtaining 20 observations from 19 studies. The REML without moderators revealed significant heterogeneity (*Q*_(19)_=34.65, *p* = 0.01, *I*^2^ = 45.1 %, tau^2^ = 0.08, SE = 0.06) but, as reported in the supplementary materials, was also the best-fit model and revealed a significant between-group difference (*Z*= −15.76, SE =0.09, *p* < 0.001; see Fig. 8, panel a, and Table 2).

#### Fluency—time limited (TL)

The initial dataset had 21 observations from 20 studies (Halliday et al. (2014) reported data from a younger and an older group of children). The best-fit model included the two moderators (see supplementary materials) from which we obtained a significant residual heterogeneity (QE_(17)_ = 40.38, *p* = 0.001, *I*^2^ = 58.1%; tau^2^ = 0.25, SE = 0.15) associated with a lack of effect of the two moderators (QM_(3)_=3.75, *p* = 0.28). Accordingly, only a significant difference between DG and CG emerged (*Z* = 10.61, SE = 0.25, *p* < 0.001). See Tables 1 and 2, and Fig. 8, panel b.

### Phonological awareness

#### Phonological manipulation

We obtained 56 observations extracted from 36 studies (Casini et al. (2018), Ruffino et al. (2014), Altarelli et al., (2013, and Lallier et al., (2013c) reported results from two experiments in the same work); after the agg procedure and the remotion of 3 outliers (Sumner et al., (2014); Anderson et al., (2013); Du & Kelly, (2013)), 37 observations from 33 studies remained.

**Fig. 9:**
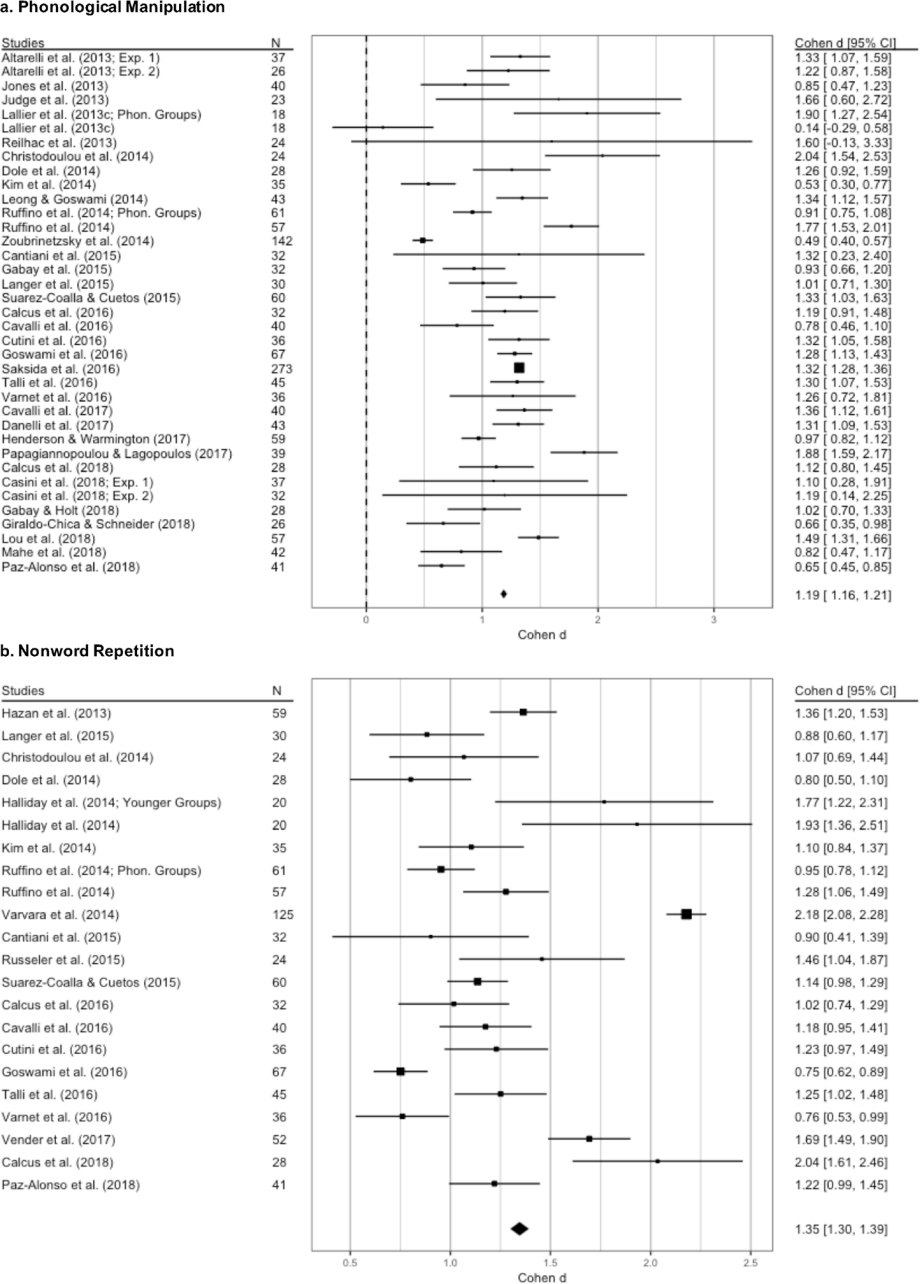
Forest plots of meta-analyses conducted on phonological awareness. The average effect size for each model is represented by the black diamond. Panel **a**: effect size of the difference between typical and dyslexic readers in phonological manipulation tasks (such as phoneme deletion and elision, segmentation and synthesis, and spoonerisms). Panel **b**: effect size of the difference between typical and dyslexic readers in nonword repetition

The best fitting model was the REML without moderators (see supplementary materials for more details). It reveals a nonsignificant level of heterogeneity (Q_(36)_ = 42.29, *p* = 0.21, *I*^2^ = 23.18%, tau^2^ = 0.03; SE = 0.03) while a significant between-groups difference emerged (*Z* = 16.40, SE = 0.06, *p* < 0.001; see Fig. 9, panel a, and Table 2).

### Nonword repetition

The initial dataset included 23 observations from 20 selected studies; Cantiani et al. (2015) particularly, reported 2 aggregated dependent measures, and the studies of Halliday et al. (2014) and Ruffino et al. (2014) reported data from two different samples. After the *agg* procedure, the resulting dataset included 22 observations.

The best fitting model was the REML without moderators (see supplementary materials for more details). It revealed a significant level of heterogeneity (Q_(21)_ = 34.67, *p* = 0.03, *I*^2^ = 39.7%, tau^2^ = 0.07; SE = 0.06), and a significant between-groups difference (*Z* = 12.94, SE = 0.09, *p* < 0.001, see Fig. 9, panel b, and Table 2 for more details).

#### RAN

The initial dataset had 25 studies that reported 50 overall observations; after merging the effect sizes from the repeated measures, 27 data points extracted from 25 studies remained (Altarelli et al. (2013), Jones et al. (2013), and Lallier et al. (2013c) reported the data of 2 independent samples for both dyslexics and controls).

**Fig. 10:**
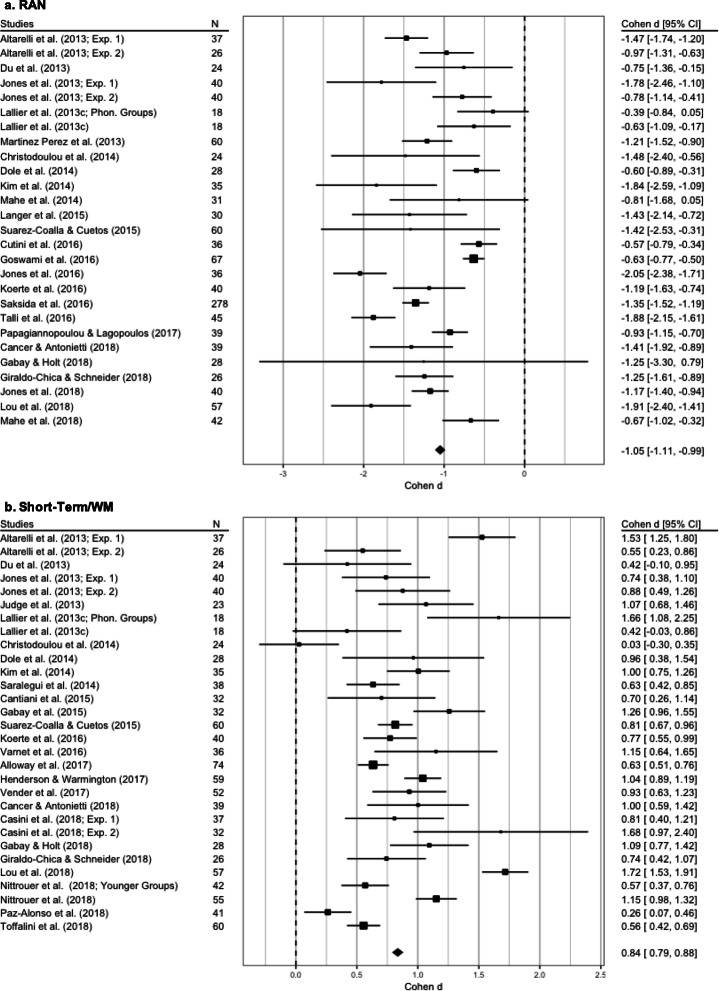
Forest plots of meta-analyses performed on rapid automatized naming (panel **a**) and short-term working memory (panel **b**). The average effect size is indicated by the black diamond

The REML without moderators showed the best fit to the data (see the supplementary materials) and revealed a nonsignificant heterogeneity (*Q*_(26)_ = 31.21, *p* = 0.22; *I*^2^ = 23.89%, tau^2^ = 0.05, SE = 0.06). A significant between-group difference emerged (Z = −11.62, SE = 0.09, *p* < 0.001; see Fig. 10 panel a, and Table 2).

#### Short-term/WM

After aggregating 10 dependent measures, we obtained 30 observations from 25 studies (Casini et al. (2018), Altarelli et al. (2013), Jones et al. (2013), Lallier et al. (2013c), and Nittrouer et al. (2018) reported results from two different samples of children for both DG and CG).

The REML without moderators showed the best data fit (see the supplementary materials), and the heterogeneity level was nonsignificant (*Q*_(29)_ = 32.27, *p* = 0.3, *I*^2^ = 15.6%, tau^2^ = 0.02, SE = 0.04). The DG and CG showed significantly different performances (*Z* = 11.6, SE = 0.07, *p* < 0.001; see Fig. 10, panel b, and Table 2).

#### Nonverbal reasoning

This dataset had 49 observations extracted from 47 papers (Lallier et al. (2013c) and Halliday et al. (2014) reported results from two different dyslexic and control samples).

**Fig. 11:**
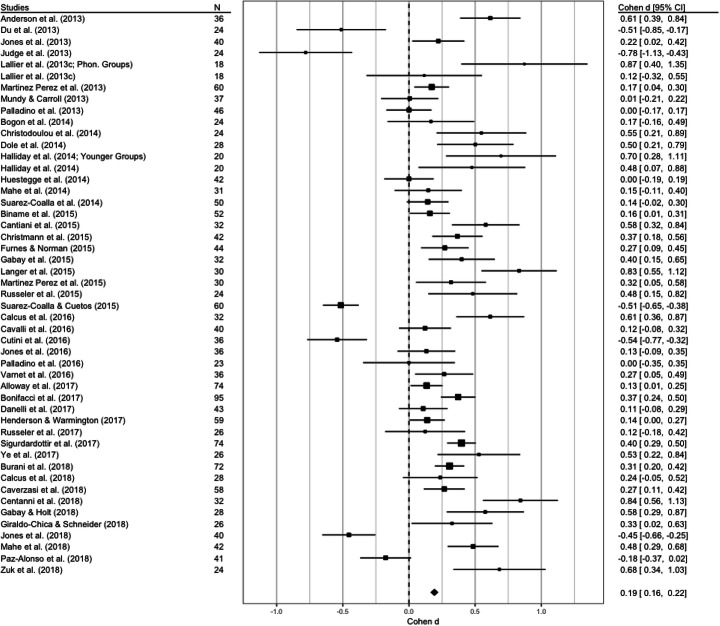
Forest plot of the meta-analysis conducted for nonverbal reasoning. The average effect size is represented by the black diamond

The tau value of the first REML was null (tau^2^ = 0; SE = 0.02); hence, we ran an FE model and obtained a better data fit (see the supplementary materials). The FE model did not show a nonsignificant level of heterogeneity (*Q*_(48)_ = 48.79, *p* = 0.44) but showed a significant between-groups difference (*Z* = 4.46, SE = 0.04, *p* < 0.001; see Fig. 11 and Table 2).

#### Nonverbal reasoning and word reading

To understand whether systematic differences in nonverbal reasoning found in DG and CG would affect reading skills, we used a meta-regression approach. Effect sizes of nonverbal reasoning were tested as moderators on all indices of word reading (Accuracy, TU, TL).

In particular, 21 studies were included for word reading accuracy, and no effect of moderators was found (QM_(1)_ = 2.44, *p* = 0.11), unless a significant degree of heterogeneity emerged by the REML (QE_(19) =_ 41.1_,_ *p* = 0.002, *I*^2^= 54.02%, tau^2^=0.18, SE = 0.11).

 A similar pattern of results emerged for both indices of word reading fluency (12 studies for TU, 15 studies for TL), in which no significant level of heterogeneity (TU: QE_(10) =_ 16.51_,_ *p* = 0.08, *I*^2^= 41.11%, tau^2^=0.1, SE = 0.11; TL: QE_(13) =_ 20.42_,_ *p* = 0.08, *I*^2^= 28.63%, tau^2^=0.07, SE = 0.09) and no significant effect of nonverbal reasoning (TU: QM_(1)_ = 0.72, *p* = 0.39; TL: QM_(1)_ = 2.98, *p* = 0.08) emerged.

## Discussion

Dyslexia research has been prolific in the last 80 years; a search of the term “developmental dyslexia” in international repositories such as PubMed retrieves a list of 10,145 studies from 1946 to 2019 (as of December 2019). With this meta-analysis, we summarized the most recent (2013–2018) behavioral findings on this topic considering both orthographic depth and a developmental perspective (operationalized as a dichotomous variable: children vs. adults). Of the many DD-associated behavioral features, we focused on two main aspects: behavioral performance in reading tasks and the main cognitive underpinnings of DD. At the cognitive level, we specifically explored PA, RAN, and short-term/WM, which are considered the main reading ability predictors (Landerl et al., 2013; Moll et al., 2014; Paulesu et al., 2001; Vaessen et al., 2010; Ziegler et al., 2010), and nonverbal reasoning, which is often used to assess the discrepancy criterion (Gustafson & Samuelsson, 1999; Goswami, 2001). Our aim was twofold: to identify DD’s universal manifestations and to determine the features of DD that can be moderated by age, orthography, or an interaction of the two at the behavioral and cognitive levels.

 We will first discuss the behavioral findings extracted by the reading tasks. Second, we will discuss the meta-analysis’ results for the cognitive dimensions of interest and, third, conclude with a general discussion, the implications for clinical practice, limitations, and future directions.

### Reading skills across ages and orthographies

The introduction stated that cognitive models of reading (Coltheart et al., 2001; Perry et al., 2010) and reading acquisition (Ehri, 1987, 1991, 1995, 2005, 2014; Ehri & McCormick, 1998; Frith, 1985; Ziegler et al., 2020) are usually based on a dual-route: a sub-lexical route implying a grapheme-to-phoneme conversion (i.e., *phonological recoding*) and a lexical route based on orthographic whole-word analysis (i.e., lexical recognition). Accordingly, phonological recoding is now widely assumed to represent the first reading strategy to be developed; in a further step, the recurrent presentations of the same words would support the development of a whole-word lexical retrieval strategy (Ehri, 2005; Grainger & Ziegler, 2011; Ziegler & Goswami, 2006). Consequently, to address both the linguistic and developmental effects on reading skills while separating lexical processing from phonological recoding, we ran separate meta-analyses for words and for the process we defined as “non-lexical decoding” (nonword/pseudoword reading), considering accuracy and fluency level (measured as either TL or TU).

Interestingly, the pattern of results was the same for the fluency indices from both word and pseudoword reading, but some intriguing differences were found for the accuracy indices. Indeed, word reading accuracy showed a systematic difference between dyslexic readers and age-matched controls that was moderated by an orthography-by-age interaction effect, while between-group differences at the fluency level were “universal,” that is, not moderated by the orthographic depth and stable at the developmental level.

As stated previously, we expected between-group differences moderated by both age and orthographic depth to represent behavioral differences between dyslexics and controls that characterize both groups’ developmental trajectories in a specific linguistic context. In particular, the significant orthography-by-age interaction effect found for word reading accuracy suggests that orthographic depth shapes the developmental trajectories of dyslexic and typical readers (Landerl et al., 1997; Ziegler et al., 2003). In deep orthographies, the average effect size between developmental dyslexic readers and age-matched controls starts from 2.4 to reach a mean Cohen’s D of 0.8 in adulthood. Shallow orthographies show a smoother developmental trajectory slope (ranging from a mean Cohen’s D of 1.5 in children to 1.1 in adulthood). These results suggest that while both typical and dyslexic readers in shallow orthographies showed a higher level of lexical decoding accuracy than children in deep orthographies, the language-specific effect in the behavioral manifestation of DD becomes less significant in adulthood. Indeed, what is more worthy to note here is that developmental trajectories are moderated by orthographic depth.

As already observed (Aro & Wimmer, 2003; Caravolas et al., 2013; Seymour et al., 2003), the acquisition of familiar-word reading seems more challenging for typical readers of deep orthographies than for their shallow counterparts. In this perspective, the gap reduction between DG and CG of deep orthographies from children to adulthood can be explained as a delayed maturation of reading efficacy by typical readers rather than as a sign of better compensation by dyslexic readers of shallow orthographies. In this sense, the differences observed in word reading accuracy between orthographies should be interpreted as a language-specific effect rather than as a feature of dyslexia.

A different pattern of results emerged for accuracy in phonological recoding, that is, non-lexical decoding. Here, the difference between deep and shallow orthographies did not emerge, while a significant difference between children and adult readers emerged: in children, the average effect size between age-matched controls and developmental dyslexics starts from 1.9 to reach1.2 in adulthood.

As Ziegler et al. (2003) suggested, a key finding of cross-linguistic studies in European languages is that children learning to read in a shallow orthography first rely on non-lexical decoding, whereas children in countries with a deep orthographic system, such as English-speaking countries, are forced to support grapheme-phoneme decoding with rhyming and whole-word strategies because of the orthographic system’s inconsistency. Nevertheless, our data suggest that DD would be characterized by an initial universal deficit in the development of phonological serial recoding during childhood, which would prevent the accurate reading of pseudowords. This deficit in phonological recoding would represent, according to the phonological theory (Bishop & Snowling, 2004; Bradley & Bryant, 1978; Démonet et al., 2004; Gabrieli, 2009; Peterson & Pennington, 2015; Vellutino et al., 2004; Snowling, 1981; Stanovich, 1988; Vellutino, 1979), a core deficit in DD, and, according to our results, is also maintained in adulthood, even though with a lesser degree of severity.

These results are in line with the idea of compensation in reading accuracy as a function of age and print exposure (Eloranta et al., 2019). In other words, reading fluency could represent a more stable marker of DD across ages than reading accuracy as, in this latter measure, a ceiling level can be more easily achieved in adulthood (Reis et al., 2020).

Indeed, more consistent findings were observed for reading speed of both word and pseudoword: no influence of orthography or age was detected for reading fluency expressed in terms of TL and TU.

According to our hypothesis, the between-group differences not moderated by age and orthography would represent the empirical manifestation of universal cognitive DD markers in line with Landerl et al. (1997) and Ziegler et al. (2003). This fluency deficit appears to similarly affect lexical decoding and phonological recoding and, according to our results, remains the most stable and reliable DD index in reading tasks. Moreover, our analysis of reading fluency needs a further methodological comment; namely, similar results emerged from the TL and TU measures of reading fluency, suggesting that the two methods are virtually identical and interchangeable. Nevertheless, the results on reading fluency, especially for models applied on time-limited measures, should be taken with *a grain of salt*: as shown in Table 2, for both word reading and Non-Lexical decoding, the number of studies in deep orthographies considerably exceed those in the shallow level. Further studies are needed to address whether this negative finding is replicated or if it represents a mere artifact owing to a lack of statistical power.

Notably, the meta-analytic approach allowed us to quantitatively support the role of reading fluency in assessing DD regardless of orthography. Beyond the above-mentioned methodological considerations, fluency measures suggest that children and adults with DD have a universal reading speed impairment. Interestingly, adults did not show any improvement in reading speed, contrary to reading accuracy, which suggests that while accuracy measures can capitalize on experience and years of reading practice, fluency measures (i.e., measures of the reading process’ automation) remain impaired. Accordingly, once again, we can conclude that the automation deficit must be considered a more reliable marker of DD.

### Cognitive predictors/markers of reading across orthographies

As described earlier, cross-linguistic studies have focused more on cognitive predictors/markers^1^ of reading skills than on reading per se. For example, Ziegler et al. (2010) tested universal reading predictors for typical Finnish, Hungarian, Dutch, Portuguese, and French second-grade readers, whereas others (Landerl et al., 2013; Moll et al., 2014; Vaessen et al., 2010) focused on PA and RAN.

Ziegler et al. (2010) suggested that PA, in terms of phoneme deletion, was a direct predictor of reading accuracy and speed in all the tested orthographies except Finnish. The authors further argued that PA, as measured by phoneme deletion, would facilitate reading irrespective of a specific country’s orthographic system, but the strength of the association between PA and reading would be moderated by the consistency of the grapheme-to-phoneme correspondence (Ziegler et al., 2010). This is consistent with our results on phonological manipulation (comprising phoneme deletion) and nonword repetition. Interestingly, although our meta-analytic approach did not allow for a direct test of the association between reading performance and phonological skills, we observed a universal PA deficit in dyslexic readers regardless of orthographic depth (consistent with Landerl et al., 2013). Nonetheless, since we observed no moderation effect of orthographic depth on PA task performance, we cannot completely support Ziegler’s assumption (Ziegler et al., 2010).

Similarly, RAN was identified as a significant reading skill predictor in all orthographies (Ziegler et al., 2010) despite the smaller effect size of this relationship compared to the one between PA and reading skills (Araújo & Faísca, 2019; Araújo et al., 2015; Kirby et al., 2010; Norton & Wolf, 2012), suggesting that RAN is a significant reading predictor albeit secondary to PA. Landerl et al.’s (2013) cross-linguistic study compared the RAN of typical and dyslexic readers; RAN was a significant marker in all the orthographies, and this was the case for both alphanumeric and picture RAN. Similarly, our meta-analysis combined data from different types of RAN and replicated Landerl et al.’s (2013) results, yet a significant level of data heterogeneity remained. This could be because of the specific materials adopted in each study and the disproportionate number of studies on shallow versus deep orthographies. Nevertheless, Araújo et al.’s (2015) meta-analysis’ results seem to support our findings and identify RAN as one of the best reading ability markers across orthographies.

Finally, notably, our results do not completely fit those of Vaessen et al. (2010). Vaessen et al. (2010) suggested that the relevance of PA and RAN in predicting reading skills would change across school grades, with PA becoming less relevant and RAN more significant over time. However, we had to exclude Hungarian, Dutch, and Portuguese studies from our meta-analyses because of their median-level orthographic depth (see Seymour’s classification), and these were the exact orthographies investigated by Vaessen et al. (2010). Moreover, we could not further test this hypothesis because we pooled all the studies involving children without considering different grades.

All we can say is that adult dyslexic readers seem equally impaired in both PA and RAN as children with DD: as in the case of reading fluency, this suggests that the diagnosis of DD is supported at every age and in every orthographic system by poor phonological knowledge and automation deficit. Moreover, our results identified short-term/WM as a cognitive marker to distinguish between dyslexic readers and typical readers in both children and adults and all types of European orthographies. However, in line with Landerl et al. (2013), short-term/WM seems to play a minor role in the entire picture, as the average difference between DDs and typical readers was smaller than 1 SD and thus less relevant than the impairments observed for PA and RAN.

Nevertheless, since dyslexia is marked by a minor but stable disadvantage in short-term/WM regardless of age and orthography, we can also claim that poor performance in WM tasks characterizes the reading disability profile. Therefore, the impact of verbal WM on DD seems overestimated, as verbal WM measures may inevitably be associated with phonological skills (see, for instance, tasks under “verbal working memory” in Peng et al.’s (2018) meta-analysis). WM and PA are considered both concurrent and combined markers of reading ability, at least in children (Knoop-van Campen et al., 2018) and represent two important components of tasks like nonword repetition and phonological manipulation. Accordingly, the conflicting results in the literature about the role of WM in DD (Gray et al., 2019; Maehler & Schuchardt, 2016; Menghini et al., 2011) could be explained by the need to separate the phonological component and consider the role of WM as is.

To conclude, our meta-analytic work seems to support both the double deficit hypothesis (Wolf & Bowers, 1999) and, more in general, to the phonological theory (Démonet et al., 2004; Vellutino et al., 2004; Bishop & Snowling, 2004).

Moreover, we included nonverbal reasoning measures in our meta-analytic procedure for two main reasons: to have a measure of intelligence (as this is a key feature of DD diagnosis) and to assess the reliability and consistency of the visuoperceptual deficit often identified as a possible DD marker (Skottun, 2000; Stein et al., 2000) across studies. Interestingly, our results suggest that while the average gap between DD and CG was 0.19 SD, a value that indicates normal performance in the nonverbal reasoning tests, we found a systematic between-group difference. This means that even within the “normal” performance range, from a psychological viewpoint, some DD-related factors as visuo-perceptual or working memory weaknesses can negatively affect the performance of dyslexic readers in reasoning tasks, through which a “lack of normality” emerges from a statistical perspective (Capitani, 1997). This should be considered when examining the “discrepancy” criterion. Nevertheless, the discrepancy criterion, when tested through a meta-regression, emerged as relevant since differences in nonverbal IQ scores of DG and CG had no significant influence in predicting their word reading outcomes (in both accuracy and fluency indices). Nonverbal reasoning, in these terms, cannot account for the word reading deficit observed for DD readers, further suggesting is independence from more general reasoning skills.

Finally, in terms of developmental level, all cognitive predictors of DD remain impaired across the entire life span regardless of orthographic level; here, PA, automation, and short-term/WM (even if with lesser strength) are all concurrent predictors of an efficient reading system.

### General discussion and some clinical remarks

This meta-analytic study attempted to summarize the most recent literature on DD from a cross-linguistic perspective and compared impaired and non-impaired children and adult readers. As a result, we obtained some relevant cross-linguistic and developmental findings useful when approaching DD from both a research and a clinical standpoint.

First, our results suggest that fluency must be considered the most relevant parameter for DD diagnosis. The disadvantage in reading speed, in lexical recognition and phonological recoding, comprises the universal manifestation of reading deficits irrespective of age and orthographic depth. Thus, the adoption of time-limited approaches in reading tasks does not provide either inconsistent or less robust evidence. As the reading process is tedious for those with DD, a good compromise would be to adopt time-limited reading tasks to avoid upsetting dyslexic readers with long and difficult reading tasks with questionable reliability and clinical validity. Nevertheless, consistent with Sprenger-Charolles et al. (2011), our results warn against adopting a solely accuracy-based reading skill evaluation. Although accuracy is clearly an important parameter, especially when assessing cross-linguistic differences in reading skills, it can easily peak because of orthographic transparency or deficit compensation (at least for this parameter) in adulthood.

Second, PA, RAN, and reading fluency are the most reliable DD cognitive markers. As suggested by numerous studies, phonology is a relevant component of the reading process associated with impairment in dyslexic readers irrespective of age and orthographic depth; consequently, a PA assessment should always be included in clinical evaluations. This suggestion reveals a relevant issue, namely, the significant lack of empirical studies that included in their cognitive batteries standardized PA measures, particularly for what concerns shallow orthographies. Moreover, regarding PA, RAN should be mandatory in clinical evaluations even though the high level of heterogeneity in our meta-analysis suggests the need to identify the optimal methodological procedures and materials for evaluating this crucial cognitive dimension.

For some short-term/WM tasks such as nonword repetition (e.g. the task of Gathercole and collagues (1994), widely used in english countries), PA is also an underpinning main component. Accordingly, to avoid the risk of systematically attributing a deficit to the wrong cognitive component and to develop a comprehensive idea of verbal short term memory ability, clinical practice should adopt digit—rather than nonword—repetition when assessing short-term memory.

Finally, the results recommend trusting the discrepancy criterion. Although we found a systematic disadvantage in nonverbal reasoning among people with DD, the pooled effect was minimal (0.19). Such a small between-group difference might depend on DD-related weakness in visuoperceptual and WM skills, rather than on a more general cognitive disadvantage. In other words, dyslexic readers’ nonverbal reasoning was nevertheless in the normal range irrespectively by age. Therefore, nonverbal reasoning skills, as supported by the results of our meta-regressions, remains a valid criterion to distinguish DD from more generic intellectual impairments.

In conclusion, the results of our meta-analysis further support cross-linguistic studies on lexical reading and phonological recoding. Despite a fluency deficit in DD across European orthographies, the accuracy measures' impairment seems strongly linked to orthographic depth. Conversely, cognitive markers of reading ability, such as PA, RAN, and short-term/WM, confirm their roles in both children and adults regardless of orthography and thus represent the universal core deficits of DD.

These results offer valuable information to researchers and clinicians. On the one hand, the results provide new research perspectives regarding possible DD treatments and the development of new diagnostic tools. On the other hand, the above-mentioned clinical considerations could also be adopted in situations where differentiating DD from other reading deficits compounded by external environmental issues can be challenging, as often observed in children with multilingual backgrounds. Indeed, identifying and diagnosing DD in these specific multilingual profiles should be based primarily on the presence of a reading fluency deficit along with poor PA and RAN performance.

### Limitations and future directions

To conclude, we would like to capitalize on some limitations of this meta-analytic study to suggest some future directions for the research agenda on DD. First, some of our results must be considered with caution because of studies’ lopsidedness across ages for deep versus shallow orthographies, particularly in the case of time-limited reading fluency and RAN measures.

Although limited to studies published between 2013 and 2018, the present study was nevertheless useful in summarizing the status quo of literature on reading and DD and in highlighting some crucial points that, from a clinical perspective, represent fundamental measures to be investigated for DD diagnosis. However, more research would help clarify how orthographic knowledge shapes phonological recoding and, through an empirical comparison, whether adult readers in deep orthographies possess better word reading accuracy than readers in shallow orthographies.

We limited our analyses to an orthographic depth level evaluation without considering the complexity, the granularity, and the entropy level considered by other classifications and approaches (e.g., Grain Size Theory; Ziegler & Goswami, 2005). In addition, future meta-analyses could go beyond our dichotomous categorization (shallow vs. deep) by including studies that cover all five levels of Seymour’s classification to provide more specific differences between orthographies.

Finally, a higher level of between-studies consistency should be reached when reporting WM-measures’ results. Although short-term/WM is widely considered a separate process, we were restricted from making this distinction herein because the studies often reported forward and backward digit span indices as composite scores.

Nonetheless, through a meta-analytic approach, we attempted to address the difficulties in conducting a cross-linguistic comparison of a wide range of cognitive abilities along the life span. This meta-analysis afforded us a direct comparison between children and adults to describe the developmental trajectories of reading and DD in shallow and deep European orthographies.

## Supplementary Information

ESM 1 (PDF 346 kb)

## Footnotes

1 With the expression predictor/marker, we want to refer to a relatively fine-grained clinical/methodological distinction. In statistical terms, the independent variables within a generalized linear model (GLM) can be referred to as “predictors.” This term from the clinical and cognitive point of view may be, anyway, misleading. Here we adopted the following distinction: predictors are those cognitive skills measured before a complete reading mastering is achieved (in principle, before the third grade of school, at least) and that can predict the level of future reading performance. Markers are those cognitive skills that can distinguish typical from dyslexic readers once the learning process of reading should be achieved.
