# Supplementary material for: Orthographic depth and developmental dyslexia: a meta-analytic study
Source: Ann Dyslexia. 2021 May 12;71(3):399–438. doi: 10.1007/s11881-021-00226-0 (PMC8458191; doi:10.1007/s11881-021-00226-0)
Supplement: Supplementary file 1 — (PDF 346 kb) [file 11881_2021_226_MOESM1_ESM.pdf]

Supplementary Materials

Orthographic depth & Developmental Dyslexia: a meta-analytic study

Supplementary Table S1. Studies included in the meta-analyses. For each single study we report the cognitive domain, the behavioural test adopted and the type of measure, the country, the sample age, the language, the level of orthographic depth and depth grade as indicated in Seymour et al. (2003).

Authors	Cognitive Domain	Measure	Test	Sample Age	Country	Language	Depth	Depth Grade
Burani et al. (2018)	Non-Verbal Reasoning		CPM	Children	Italy	Italian	Shallow	2
Calcus et al., (2018)	Word reading	Accuracy	BELEC	Children	Belgium	French	Deep	4
	Non-lexical decoding	Accuracy	BELEC	Children				
	Phonological Awareness (Manipulation & STM)	Accuracy	BELEC	Children				
	Non-Verbal Reasoning		Wechsler Non Verbal scale of ability (Wechsler, 1996)	Children				
Cancer & Antonietti (2018)	RAN	Time	Rapid Naming Test and Visual Search of Colours, Figures, and Numbers	Adults	Italy	Italian	Shallow	2
	Short-Term/WM	Digit span Forward, Digit Span Backward	WAIS-R	Adults				
Casini et al. (2018)	Word reading	Accuracy	L2MA	Children	France	French	Deep	4
	Word reading	Time Limited	LUM	Children				
	Phonological Awareness (Manipulation)	Accuracy	(Sprenger-Charolles et al. 2005)	Children				
	Short-Term/WM	Digit span Forward, Digit Span Backward		Children				
Caverzasi et al. (2018)	Word reading	Time Limited	TOWRE	Children	USA	English	Deep	5
	Non-Lexical Decoding	Time Limited	TOWRE	Children				
	Non-Verbal Reasoning		WASI	Children				
Centanni et al. (2018)	Non-Lexical Decoding	Time Limited	TOWRE-PDE	Children	USA	English	Deep	5
	Non-verbal Reasoning		RIAS	Children				
Gabay & Holt (2018)	Word reading	Time Limited	TOWRE-II	Adults	USA	English	Deep	5
	Non-Lexical Decoding	Time Limited	TOWRE-II	Adults				

Authors	Cognitive Domain	Measure	Test	Sample Age	Country	Language	Depth	Depth Grade
	Phonological Awareness (Manipulation)	Accuracy	Brunswick, McCrory, Price, Frith, Frith (1999)	Adults				
	RAN	Accuracy	Denkla & Rudel (1976)	Adults				
	Short-Term/WM	Digit span Forward and Backward	WAIS-III	Adults				
	Non-Verbal Reasoning		SPM	Adults				
Giraldo-Chica & Schneider (2018)	Non-Lexical Decoding	Accuracy	WJ III	Adults	USA	English	Deep	5
	Phonological Awareness (Manipulation)	Accuracy	CTOPP	Adults				
	RAN	Time	CTOPP	Adults				
	Short-Term/WM	Digit span Forward and Backward	WAIS-III	Adults				
	Non-Verbal Reasoning		WAIS-III	Adults				
Jones et al. (2018)	Word reading	Accuracy, Time Limited	TOWRE	Adults	UK	English	Deep	5
	Non-Lexical Decoding	Accuracy, Time Limited	TOWRE	Adults				
	RAN	Time	CTOPP	Adults				
	Non-Verbal Reasoning		WASI	Adults				
Lou et al. (2018)	Word reading	Accuracy	ODEDYS	Children	France	French	Deep	4
	Phonological Awareness (Manipulation)	Accuracy	Sprenger Charolles et al. (2005)	Children				
	RAN	Time	Plaza & Robert-Jahier, (2006)	Children				
	Short-Term/WM	Digit span Forward and Backward	WISC-IV	Children				
Mahé et al. (2018)	Word reading	Accuracy, Time Unlimited	ECLA+16	Adults	France	French	Deep	4
	Non-Lexical Decoding	Accuracy, Time Unlimited	ECLA+16	Adults				
	Phonological Awareness (Manipulation)	Accuracy	ECLA+16	Adults				
	RAN	Time	Evalad Battery	Adults				
	Non-Verbal Reasoning		SPM	Adults				

Authors	Cognitive Domain	Measure	Test	Sample Age	Country	Language	Depth	Depth Grade
Nittrouer et al. (2018)	Short-Term/WM	Forward Digit Span	WISC-IV	Children	USA	English	Deep	5
Paz-Alonso et al. (2018)	Word reading	Accuracy, Time Unlimited	PROLEC	Adults	Spain	Spanish	Shallow	2
	Non-Lexical Decoding	Accuracy, Time Unlimited	PROLEC	Adults				
	Phonological Awareness (Manipulation & STM)	Accuracy		Adults				
	Short-Term/WM	Working Memory Span	WISC-R	Adults				
	Non-Verbal Reasoning		WISC-R	Adults				
Toffalini et al. (2018)	Non-Lexical Decoding	Accuracy, Time Unlimited	DDE-2	Children	Italy	Italian	Shallow	2
	Short-Term/WM	Forward Digit Span	WISC-IV	Children				
Zuk et al. (2018)	Word reading	Time Limited	TOWRE	Children	USA	English	Deep	5
	Non-Verbal Reasoning		K-BIT	Children				
Alloway et al. (2017)	Short-Term/WM	Verbal Working Memory	AWMA	Children	UK	English	Deep	5
	Non-Verbal Reasoning		WASI	Children				
Bonifacci et al. (2017)	Word reading	Time Limited	ALCE	Children	Italy	Italian	Shallow	2
	Non-Lexical Decoding	Time Limited	ALCE	Children				
	Non-Verbal Reasoning		Thurstone & Thurstone (1963, 1981)	Children				
Cavalli et al. (2017)	Non-Lexical Decoding	Accuracy,	Sprenger-Charolles et al. (2005)	Adults	France	French	Deep	4
	Phonological Awareness (Manipulation & STM)	Accuracy	Sprenger-Charolles et al. 2005	Adults				
Danelli et al. (2017)	Word reading	Accuracy, Time Unlimited	Paulesu et al. (2001)	Adults	Italy	Italian	Shallow	2
	Non-Lexical Decoding	Accuracy	Paulesu et al. (2001)	Adults				
	Phonological Awareness (Manipulation)	Accuracy	Paulesu et al. (2001)	Adults				
	Non-Verbal Reasoning		WAIS-R	Adults				
Henderson & Warmington (2017)	Word reading	Time Limited	TOWRE	Adults	UK	English	Deep	5

Authors	Cognitive Domain	Measure	Test	Sample Age	Country	Language	Depth	Depth Grade
	Non-Lexical Decoding	Time Limited	TOWRE	Adults				
	Phonological Awareness (Manipulation)	Accuracy	CTOPP	Adults				
	Short-Term/WM	Verbal Working Memory	AWMA	Adults				
	Non-Verbal Reasoning		WASI	Adults				
Papagiannopoulou & Lagopoulos (2017)	Word reading	Time Limited	One minute reading	Children	Australia	English	Deep	5
	Phonological Awareness (Manipulation)	Accuracy		Children				
	RAN	Time	DEST/DST-J	Children				
Rüsseler et al. (2017)	Word reading	Accuracy		Adults	Germany	German	Shallow	2
	Non-Lexical Decoding	Accuracy						
	Non-Verbal Reasoning		WAIS	Adults				
Sigurdardottir et al. (2017)	Word reading	Accuracy, Time Limited	IS-FORM	Adults	Iceland	Icelandic	Shallow	2
	Non-Lexical Decoding	Accuracy, Time Limited	IS-PSEUDO	Adults				
	Non-Verbal Reasoning		WAIS	Adults				
Vender et al. (2017)	Word reading	Accuracy, Time Unlimited	DDE-2	Children	Italy	Italian	Shallow	2
	Non-Lexical Decoding	Accuracy,	DDE-2	Children				
	Phonological Awareness (STM)	Accuracy	Stella & Job (2001)	Children				
	Short-Term/WM	Digit span Forward, Digit Span Backward	Memory Battery For Children	Children				
Ye et al. (2017)	Word reading	Accuracy		Adults	Germany	German	Shallow	2
	Non-Lexical Decoding	Accuracy		Adults				
	Non-Verbal Reasoning		WAIS	Adults				
Calcus et al., (2016)	Word reading	Accuracy	BELEC	Children	Belgium	French	Deep	4
	Phonological Awareness (Manipulation & STM)	Accuracy	BELEC	Children				

Authors	Cognitive Domain	Measure	Test	Sample Age	Country	Language	Depth	Depth Grade
	Non-Verbal Reasoning		Wechsler Nonverbal Scale of Ability (Wechsler, 1996).	Children				
Cavalli et al. (2016)	Word reading	Accuracy, Time Unlimited	EVALEC	Adults	France	French	Deep	4
	Non-Lexical Decoding	Accuracy, Time Unlimited	EVALEC	Adults				
	Phonological Awareness (Manipulation & STM)	Accuracy	EVALEC	Adults				
	Non-Verbal Reasoning		SPM	Adults				
Cutini et al. (2016)	Phonological Awareness (Manipulation & STM)	Accuracy	Southern British English	Children	UK	English	Deep	5
	RAN	Time	Richardson et al. (2004)	Children				
	Non-Verbal Reasoning		WISC-III	Children				
Farris et al. (2016)	Word reading	Accuracy, Time Limited	WRMT	Children	USA	English	Deep	5
	Non-Lexical Decoding	Accuracy, Time Limited	WRMT	Children				
Ghisi et al. (2016)	Word reading	Accuracy, Time Limited	DDE-2	Adults	Italy	Italian	Shallow	2
	Non-Lexical Decoding	Accuracy, Time Limited	DDE-2	Adults				
Goswami et al. (2016)	Phonological Awareness (Manipulation & STM)	Accuracy	Thomson & Goswami (2008)	Children	UK	English	Deep	5
	RAN	Time	Kuppeen et al. (2011)	Children				
Jones et al. (2016)	Word reading	, Time Unlimited	TOWRE	Adults	UK	English	Deep	5
	Non-Lexical Decoding	Accuracy, Time Unlimited	TOWRE	Adults				
	RAN	Time	CTOPP	Adults				
	Non-Verbal Reasoning		WASI	Adults				
Koerte et al. (2016)	Word reading	Time Limited	Salzburger Reading and Spelling Test	Children	Germany	German	Shallow	2
	Non-Lexical Decoding	Time Limited	Salzburger Reading and Spelling Test	Children				
	RAN	Accuracy/Time Limited	Denckla & Rudel (1976)	Children				
	Short-Term/WM	Digit span Forward and Backward	WISC-IV	Children				

Authors	Cognitive Domain	Measure	Test	Sample Age	Country	Language	Depth	Depth Grade
Palladino et al. (2016)	Non-Verbal Reasoning		CPM	Children	Italy	Italian	Shallow	2
Saksida et al. (2016)	Word reading	Accuracy, Time Unlimited	ODEDYS	Children	France	French	Deep	4
	Non-Lexical Decoding	Accuracy, Time Unlimited	ODEDYS	Children				
	Phonological Awareness (Manipulation)	Accuracy	Sprenger-Charolles et al. (2005)	Children				
	RAN	Time		Children				
Talli et al. (2016)	Word reading	Time Limited	Hatzigeorgiou et al. (2000)	Children	Greece	Greek	Shallow	2
	Non-Lexical Decoding	Time Limited	Protopapas et al. (2012)	Children				
	Phonological Awareness (Manipulation & STM)	Accuracy	EVALEC	Children				
	RAN	Time	Vellutino et al. (2004)	Children				
Varnet et al. (2016)	Word reading	Accuracy, Time Unlimited	ECLA+16	Adults	France	French	Deep	4
	Non-Lexical Decoding	Accuracy, Time Unlimited	ECLA+16	Adults				
	Phonological Awareness (Manipulation & STM)	Accuracy	ECLA+16	Adults				
	Short-Term/WM	Digit span Forward and Backward	ECLA+16	Adults				
	Non-Verbal Reasoning		SPM	Adults				
Binamé et al. (2015)	Non-Verbal Reasoning		SPM	Children	France	French	Deep	4
Cantiani et al. (2015)	Phonological Awareness (Manipulation & STM)	Accuracy	VAUMeLF	Children	Italy	Italian	Shallow	2
	Short-Term/WM	Digit span Forward and Backward	Bisiacchi et al. (2005)	Children				
	Non-Verbal Reasoning		Cattell (1979)	Children				
Christmann et al. (2015)	Word reading	Accuracy, Time Unlimited	Schulte-Körne, (2001)	Adults	Germany	German	Shallow	2
	Non-Lexical Decoding	Accuracy, Time Unlimited	Schulte-Körne, (2001)	Adults				
	Non-Verbal Reasoning		CFT-20-R	Adults				
Furnes & Norman (2015)	Non-Verbal Reasoning		(Naglieri, 2008)	Adults	Norway	Norwegian	Shallow	2
Gabay et al. (2015)	Word reading	Time Limited	TOWRE	Adults	USA	English	Deep	5

Authors	Cognitive Domain	Measure	Test	Sample Age	Country	Language	Depth	Depth Grade
	Non-Lexical Decoding	Accuracy	WRMT-R	Adults				
	Non-Lexical Decoding	Time Limited	TOWRE	Adults				
	Phonological Awareness (Manipulation)	Accuracy	Brunswick et al. (1999)	Adults				
	Short-Term/WM	Forward Digit Span	WAIS	Adults				
	Non-Verbal Reasoning		SPM	Adults				
Langer et al. (2015)	Word reading	Time Limited	TOWRE	Children	USA	English	Deep	5
	Non-Lexical Decoding	Time Limited	TOWRE	Children				
	Phonological Awareness (Manipulation & STM)	Accuracy	CTOPP	Children				
	RAN	Accuracy	RAN/RAS	Children				
	Non-Verbal Reasoning		K-BIT	Children				
Martinez Perez et al. (2015)	Word reading	Time Unlimited		Adults	France	French	Deep	4
	Non-Verbal Reasoning		SPM	Adults				
Moores et al. (2015)	Word reading	Accuracy	PALPA	Adults	UK	English	Deep	5
	Non-Lexical Decoding	Accuracy	PALPA	Adults				
Rüsseler et al. (2015)	Word reading	Accuracy	Rüsseler et al. (2003)	Adults	Germany	German	Shallow	2
	Non-Lexical Decoding	Accuracy	Rüsseler et al. (2003)	Adults				
	Phonological Awareness (STM)	Accuracy	ZTL Mottier Test subtest	Adults				
	Non-Verbal Reasoning		WAIS-R	Adults				
Suárez-Coalla & Cuetos (2015)	Word reading	Accuracy, Time Unlimited	Ramos & Cuetos, (2005)	Adults	Spain	Spanish	Shallow	2
	Non-Lexical Decoding	Accuracy	Ramos & Cuetos, (2005)	Adults				
	Phonological Awareness (Manipulation & STM)	Accuracy		Adults				
	RAN	Time		Adults				
	Short-Term/WM	Digit span Forward and Backward	WAIS-R	Adults				
	Non-Verbal Reasoning		WAIS-R	Adults				

Authors	Cognitive Domain	Measure	Test	Sample Age	Country	Language	Depth	Depth Grade
Asbjørnsen et al. (2014)	Non-Lexical Decoding	Accuracy	WJ-III	Children	USA	English	Deep	5
Bogon et al. (2014)	Word reading	Time Limited	One Minute Reading Fluency Test	Children	Germany	German	Shallow	2
	Non-Lexical Decoding	Time Limited	One Minute Reading Fluency Test	Children				
	Non-Verbal Reasoning		CFT-20-R	Children				
Christodoulou et al. (2014)	Word reading	Accuracy, Time Limited	WRMT	Adults	USA	English	Deep	5
	Non-Lexical Decoding	Accuracy	WRMT	Adults				
	Phonological Awareness (Manipulation & STM)	Accuracy	CTOPP	Adults				
	RAN	Accuracy	RAN/RAS	Adults				
	Short-Term/WM	Memory for digits	CTOPP	Adults				
	Non-Verbal Reasoning		WASI	Adults				
Dole et al. (2014)	Word reading	Accuracy, Time Unlimited	Alouette & ODEDYS	Adults	France	French	Deep	4
	Non-Lexical Decoding	Accuracy, Time Unlimited	ODEDYS	Adults				
	Phonological Awareness (Manipulation & STM)	Accuracy	ODEDYS	Adults				
	RAN	Time	ODEDYS	Adults				
	Short-Term/WM	Digit span Forward and Backward	ODEDYS	Adults				
	Non-Verbal Reasoning		SPM	Adults				
Halliday et al. (2014)	Word reading	Time Limited	TOWRE	Children	UK	English	Deep	5
	Non-Lexical Decoding	Time Limited	TOWRE	Children				
	Phonological Awareness (STM)	Accuracy	NEPSY	Children				
	Non-Verbal Reasoning		WASI	Children				
Huestegge et al. (2014)	Non-Verbal Reasoning		CFT-20-R	Children	Germany	German	Shallow	2
Kim et al. (2014)	Word reading	Time Limited	TOWRE	Adults	USA	English	Deep	5
	Non-Lexical Decoding	Time Limited	TOWRE	Adults				
	Phonological Awareness (Manipulation & STM)	Accuracy	CTOPP	Adults				

Authors	Cognitive Domain	Measure	Test	Sample Age	Country	Language	Depth	Depth Grade
	RAN	Accuracy	CTOPP	Adults				
	Short-Term/WM	Memory for digits	CTOPP	Adults				
Leong & Goswami (2014)	Word reading	Accuracy	WRAT-III	Adults	UK	English	Deep	5
	Phonological Awareness (Manipulation)	Accuracy	PhAB	Adults				
Mahé et al. (2014)	RAN	Time	Phonological Assessment Battery	Adults	France	French	Deep	4
	Non-Verbal Reasoning		SPM	Adults				
Perea et al. (2014)	Word reading	Accuracy, Time Unlimited	PROLEC-R	Children	Spain	Spanish	Shallow	2
	Non-Lexical Decoding	Accuracy, Time Unlimited	PROLEC-R	Children				
Ruffino et al. (2014)	Word reading	Accuracy	DDE-2	Children	Italy	Italian	Shallow	2
	Non-Lexical Decoding	Accuracy	DDE-2	Children				
	Phonological Awareness (Manipulation & STM)	Accuracy		Children				
Saralegui et al. (2014)	Word reading	Accuracy, Time Unlimited	PROLEC-R	Children	Spain	Spanish	Shallow	2
	Non-Lexical Decoding	Accuracy, Time Unlimited	PROLEC-R	Children				
	Short-Term/WM	Digit span Forward and Backward	WISC-IV	Children				
Suarez-Coalla et al. (2014)	Word reading	Accuracy, Time Unlimited	PROLEC-R	Children	Spain	Spanish	Shallow	2
	Non-Lexical Decoding	Accuracy, Time Unlimited	PROLEC-R	Children				
	Non-Verbal Reasoning		WISC	Children				
Sumner et al. (2014)	Phonological Awareness (Manipulation)	Accuracy	Screening Test Junior	Children	UK	English	Deep	5
Varvara et al. (2014)	Phonological Awareness (STM)	Accuracy	Marotta, 2008	Children	Italy	Italian	Shallow	2
Zoubrinetzsky et al. (2014)	Word reading	Accuracy, Time Unlimited	ODEDYS	Children	France	French	Deep	4
	Non-Lexical Decoding	Accuracy, Time Unlimited	ODEDYS	Children				
	Phonological Awareness (Manipulation)	Accuracy	BELEC	Children				

Authors	Cognitive Domain	Measure	Test	Sample Age	Country	Language	Depth	Depth Grade
Altarelli et al. (2013)	Phonological Awareness (Manipulation)	Accuracy	Sprenger-Charolles et al. (2005)	Children	France	French	Deep	4
	RAN	Time	Plaza & Robert-Jahier, (2006)	Children				
	Short-Term/WM	Digit span Forward and Backward	WISC	Children				
Anderson et al. (2013)	Word reading	Accuracy	WJ III	Children	USA	English	Deep	5
	Non-Lexical Decoding	Accuracy	WJ III	Children				
	Phonological Awareness (Manipulation)	Accuracy	Gottardo et al. (2001)	Children				
	Non-Verbal Reasoning		Naglieri (1985)	Children				
Du & Kelly (2013)	Phonological Awareness (Manipulation)	Accuracy	Perin, 1983		UK	English	Deep	5
	RAN	Time	Denckla & Rudel, (1976)	Adults				
	Short-Term/WM	Verbal Working Memory	AWMA	Adults				
	Non-Verbal Reasoning		WASI	Adults				
Hazan et al. (2013)	Word reading	Time Limited	TOWRE	Children	UK	English	Deep	5
	Non-Lexical Decoding	Time Limited	TOWRE	Children				
	Phonological Awareness (STM)	Accuracy	CNRep	Children				
Jones et al. (2013)	Word reading	Accuracy	WRAT	Adults	UK	English	Deep	5
	Non-Lexical Decoding	Accuracy	PATSy	Adults				
	Phonological Awareness (Manipulation)	Accuracy	Hatcher et al. (2002)	Adults				
	RAN	Time	Denckla & Rudel, (1976)	Adults				
	Short-Term/WM	Digit span Forward and Backward	Miles, (1993)	Adults				
	Non-Verbal Reasoning		WAIS III	Adults				
Judge et al. (2013)	Word reading	Accuracy	WRAT	Adults	UK	English	Deep	5
	Non-Lexical Decoding	Accuracy	WRMT	Adults				
	Phonological Awareness (Manipulation)	Accuracy	Judge et al. (2007)	Adults				
	Short-Term/WM	Forward Digit Span	WAIS III	Adults				

Authors	Cognitive Domain	Measure	Test	Sample Age	Country	Language	Depth	Depth Grade
	Non-Verbal Reasoning		WASI	Adults				
Koyama et al. (2013)	Word reading	Time Limited	TOWRE	Children	USA	English	Deep	5
	Non-Lexical Decoding	Time Limited	TOWRE	Children				
Lallier et al. (2013a)	Word reading	Accuracy, Time Unlimited	ODEDYS	Children	France	French	Deep	4
	Non-Lexical Decoding	Accuracy, Time Unlimited	ODEDYS	Children				
Lallier et al. (2013b)	Word Reading	Accuracy, Time Unlimited	ODEDYS	Children	France	French	Deep	4
	Non-Lexical Decoding	Accuracy, Time Unlimited	ODEDYS	Children				
Lallier et al. (2013c)	Word Reading	Time Limited	DAST	Adults	UK	English	Deep	5
	Phonological Awareness (Manipulation)	Accuracy	DAST	Adults				
	RAN	Accuracy	DAST	Adults				
	Short-Term/WM	Backward Digit Span	DAST	Adults				
	Non-Verbal Reasoning		WAIS III	Adults				
Martinez Perez et al. (2013)	Word reading	Accuracy, Time Unlimited	Poncelet (1999)	Adults	Belgium	French	Deep	4
	Non-Lexical Decoding	Accuracy, Time Unlimited	Poncelet (1999)	Adults				
	RAN	Time	Phonological Assessment Battery	Adults				
	Non-Verbal Reasoning		SPM	Adults				
Mundy & Carroll (2013)	Word reading	Time Limited	TOWRE	Adults	UK	English	Deep	5
	Non-Lexical Decoding	Time Limited	TOWRE	Adults				
	Non-Verbal Reasoning		WASI	Adults				
Nittrouer & Lowenstein (2013)	Word reading	Accuracy	WRAT	Children	USA	English	Deep	5
Palladino et al. (2013)	Non-Verbal Reasoning		SPM	Children	Italy	Italian	Shallow	2
Reilhac et al. (2013)	Word reading	Accuracy, Time Unlimited	Alouette	Adults	France	French	Deep	4
	Non-Lexical Decoding	Accuracy, Time Unlimited	ODEDYS	Adults				

Authors	Cognitive Domain	Measure	Test	Sample Age	Country	Language	Depth	Depth Grade
	Phonological Awareness (Manipulation)	Accuracy	(Paulesu et al. 2001)	Adults				
Zhang et al. (2013)	Word reading	Accuracy, Time Limited	WRMT	Adults	USA	English	Deep	5
	Non-Lexical Decoding	Accuracy, Time Limited	WRMT	Adults				

Supplementary Table S2: For each single meta-analysis Random Effect Models (REMLs), without and with moderators, and a Fixed Effect Model (FE) were run. In what follows Aikake Information Criterion (AIC) values are reported. Model selection was made on the basis of the AIC value.

**Meta-analysis**	**Model**	**AIC**
Word Reading Accuracy	REML without moderators	91.86
	REML with moderators	**70.86**
	FE	tau ≠ 0*
Word Reading TU	REML without moderators	**28.9**
	REML with moderators	31.69
	FE	tau ≠ 0*
Word Reading TL	REML without moderators	**35.68**
	REML with moderators	38.17
	FE	tau ≠ 0*
Non-Lexical Decoding Accuracy	REML without moderators	66.44
	REML with moderators	**54.75**
	FE	tau ≠ 0*
Non-Lexical Decoding TU	REML without moderators	**24.37**
	REML with moderators	28.24
	FE	tau ≠ 0*
Non-Lexical Decoding TL	REML without moderators	46.06
	REML with moderators	**45.38**
	FE	tau ≠ 0*
Phonological Manipulation	REML without moderators	**43.87**
	REML with moderators	47.94
	FE	tau ≠ 0*
Nonword Repetition	REML without moderators	**28.26**
	REML with moderators	30.37
	FE	tau ≠ 0*
RAN	REML without moderators	**41.78**
	REML with moderators	43.53
	FE	tau ≠ 0*
Short-Term/WM	REML without moderators	**34.43**
	REML with moderators	37.09
	FE	tau ≠ 0*
Non-Verbal Reasoning	REML without moderators	38.30
	REML with moderators	42.88
	FE	**35.92**

*The Fixed Effect Model (FE) was not run in the case of tau ≠ 0 because the absence of data heterogeneity could not be assumed.

Supplementary Table S3: For each single meta-analysis the number of studies stratified for Orthographic Depth (Shallow vs. Deep) and Age (Children vs. Adults) is reported, together with the average ES of the between-group (dyslexic vs. controls readers) differences of age, in terms of years. To verify whether differences of age, moderated by the orthography or the age-group can be found, the orthography-by-age interaction effect was tested with a REML on each cognitive dimension. The P-value of the interaction effect is reported in the table.

	Word Reading						
	**Orthographic Depth**	**Age**	**Records (k)**	**Average ES of age**	**min.**	**max.**	**Interaction Effect (p-value)**
Accuracy	Shallow	Children	6	-0.35	-0.43	-0.28	0.32
		Adults	9	-0.13	-0.20	-0.06	
	Deep	Children	13	-0.18	-0.20	-0.16	
		Adults	13	-0.17	-0.23	-0.11	
Fluency (TU)	Shallow	Children	3	-0.38	-0.5	-0.27	0.57
		Adults	4	-0.03	-0.12	0.05	
	Deep	Children	4	-0,19	-0,22	-0.16	
		Adults	8	-0.08	-0.16	-0.01	
Fluency (TL)	Shallow	Children	3	0.07	-0.16	0.03	0.22
		Adults	1*				
	Deep	Children	11	0.01	-0.06	0.08	
		Adults	8	-0.33	-0.4	-0.25	
	Non-Lexical Decoding						
	**Orthographic Depth**	**Age**	**Records (k)**	**Average ES of age**	**min.**	**max.**	**Interaction Effect (p-value)**
Accuracy	Shallow	Children	6	-0.29	-0.36	0.22	0.51
		Adults	8*	0.13	-0.20	0.06	
	Deep	Children	7	-0.19	-0.21	-0.16	
		Adults	16	-0.14	-0.20	-0.09	
Fluency (TU)	Shallow	Children	5	-0.36	-0.43	-0.28	0.78
		Adults	3	-0.03	-0.12	0.05	
	Deep	Children	4	-0.19	-0.24	-0.18	
		Adults	8	-0.08	-0.15	0.01	
Fluency (TL)	Shallow	Children	4	0.06	-0.15	0.03	0.19

	Orthographic Depth	Age	Records (k)	Average ES of age	min.	max.	Interaction Effect (p-value)
		Adults	1*				
	Deep	Children	8	0.21	-0.1	0,.06	
		Adults	7	-0.31	-0.39	0.22	
	Phonological Awareness						
	**Orthographic Depth**	**Age**	**Records (k)**	**Average ES of age**	**min.**	**max.**	**Interaction Effect (p-value)**
Phonogical Manipulation	Shallow	Children	4	0.02	-0.07	0.11	0.96
		Adults	2*	0.02	-0.08	0.13	
	Deep	Children	13	-0.18	-0.21	-0.16	
		Adults	17	-0.12	-0.18	-0.06	
Nonword repetition	Shallow	Children	5*	-0.22	-0.30	-0.13	0.25
		Adults	3	0.05	-0.05	0.15	
	Deep	Children	7*	-0.03	-0.1	-0.05	
		Adults	5	-0.06	-0.17	0.04	
	Other Cognitive Skills						
	**Orthographic Depth**	**Age**	**Records (k)**	**Average ES of age**	**min.**	**max.**	**Interaction Effect (p-value)**
RAN	Shallow	Children	2	0.18	0.04	0.32	0.68
		Adults	2	0.19	0.08	0.30	
	Deep	Children	8	-0.19	-0.22	-0.16	
		Adults	15	-0-20	-0.26	-0.14	
Short-Term/WM	Shallow	Children	5	-0.22	-0.31	-0.14	0.35
		Adults	3	0.14	0.04	0.23	
	Deep	Children	8	-0.27	-0.34	-0.21	
		Adults	14	-0.15	-0.22	-0.09	
Non-Verbal Reasoning	Shallow	Children	8	-0.08	-0.14	0.02	0.54
		Adults	9	0.03	-0.04	0.11	
	Deep	Children	12	-0.25	-0.31	-0.18	
		Adults	20	-0.19	-0.24	-0.14	

*one missing observation due to incomplete information (SD for age was not reported)
